# Integrated transcriptomic and metabolomic analyses identify key metabolites and genes in sepsis-associated acute lung injury

**DOI:** 10.3389/fimmu.2026.1959223

**Published:** 2026-10-19

**Authors:** He Huang, Lei Zhao, He Chen, Ling Sui, Jinghe Zhu, Xiaonan Fang, Pengzhuo Li, He Liu, Xiaolin Wu, Decheng Wang

**Affiliations:** 1 Medical Inspection Center, The Second Hospital Affiliated to Liaoning University of Traditional Chinese Medicine, Shenyang, Liaoning, China; 2 Pharmacological Laboratory, Liaoning Provincial Academy of Traditional Chinese Medicine, Shenyang, Liaoning, China; 3 Animal Experimental Center, Liaoning Provincial Academy of Traditional Chinese Medicine, Shenyang, Liaoning, China

**Keywords:** oleoylcarnitine, sepsis-associated acute lung injury, T cells, untargeted metabolomics, Xuanbai Chengqi Decoction

## Abstract

**Introduction:**

Sepsis-associated acute lung injury (SALI) is a life-threatening pulmonary complication of sepsis with high mortality. This study aimed to prioritize serum metabolites and genes potentially involved in SALI by integrating multi-omics datasets.

**Methods:**

A rat SALI model treated with Xuanbai Chengqi Decoction (XCD) was subjected to untargeted serum metabolomics. Bulk and single-cell RNA-seq datasets were retrieved from GEO, and metabolite-related GWAS statistics were obtained from IEU OpenGWAS. scPagwas mapped metabolite genetic signals to cell populations; hdWGCNA identified cell signature genes. Candidate genes were filtered by overlapping bulk DEGs, ten machine-learning models and expression validation. Functional enrichment, immune infiltration, cell -cell communication, pseudotime and scTenifoldKnk virtual knockdown analyses were performed.

**Results:**

Oleoylcarnitine showed the strongest genetic association with T cells. Cross-platform integration prioritized GIMAP4 and RCBTB2 as hub genes, with WBP2 retained for further validation. Their expression correlated with altered immune infiltration, especially eosinophils, and the three-gene profiles shared 62 biological processes. SALI exhibited increased number and strength of cell interactions compared with isolated sepsis. Five T-cell subsets were identified, and virtual knockdown of hub genes modified regulation of eight DEGs.

**Discussion:**

Oleoylcarnitine-associated metabolic changes are correlated with T-cell dysregulation mediated by GIMAP4, RCBTB2, and WBP2 in SALI. These findings are descriptive, and further experiments are required to establish causal relationships.

## Graphical Abstract

**Figure:**
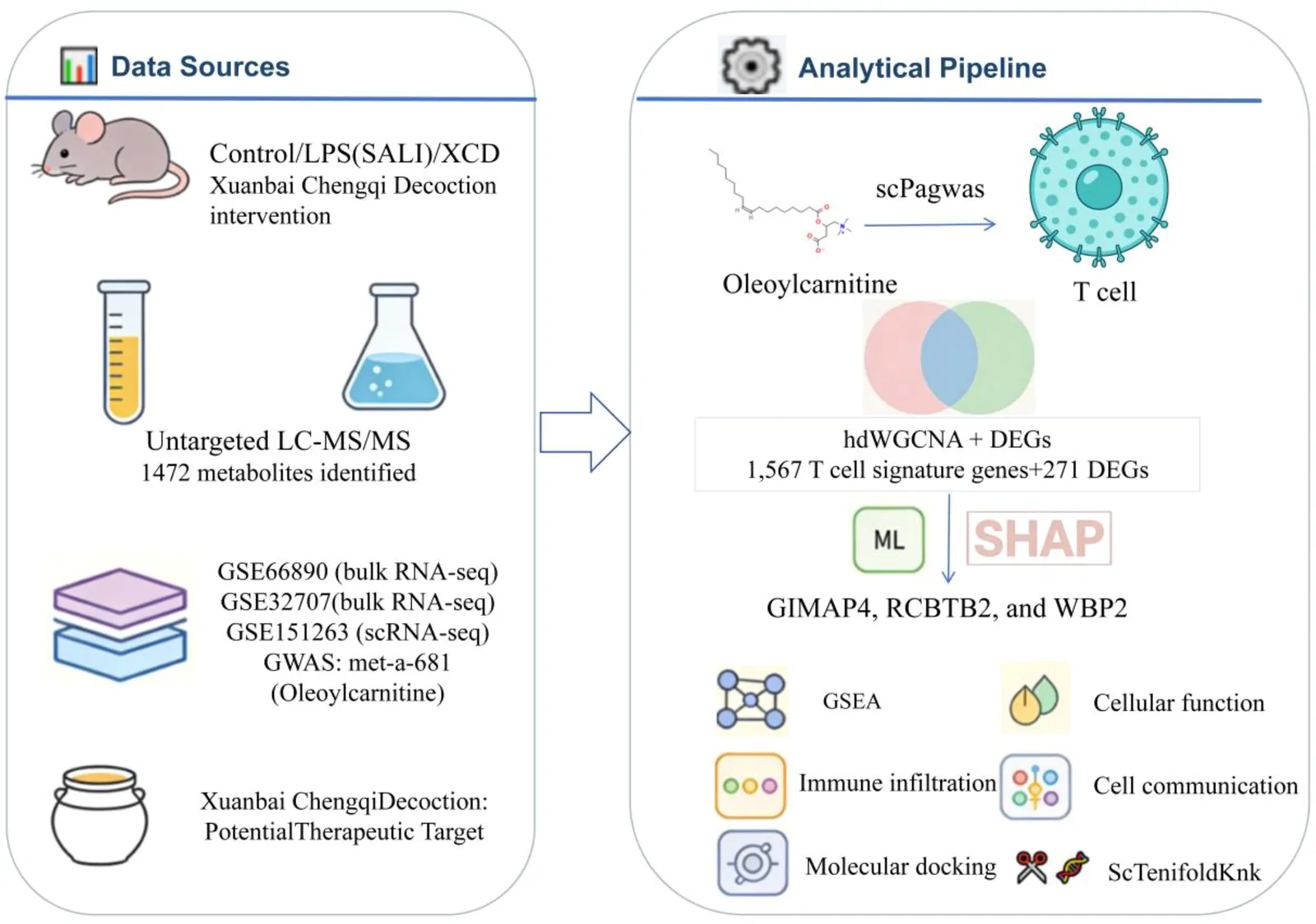

## 1 Introduction

Sepsis-associated acute lung injury (SALI) is a frequent and life-threatening complication of sepsis and may progress to acute respiratory distress syndrome (ARDS) (1), which has a reported 90-day mortality rate of 35.5–55.7% (1) and represents one of the most prevalent and life-threatening complications of sepsis (2, 3). The pathophysiology of SALI involves excessive inflammation, immune dysregulation, and metabolic imbalance, which result in resistance to neutrophil apoptosis, disruption of the alveolar-capillary barrier (4), systemic inflammatory response syndrome, disseminated intravascular coagulation (1), and respiratory failure (4). Traditional treatments have limitations such as antibiotic resistance and fluid overload; therefore, there is an urgent need for precise therapies targeting the coordinated mechanism of inflammation and apoptosis (2, 3, 5).

Research on SALI has focused on inflammatory factors (e.g., TNF-α, IL-6), immune cells (e.g., macrophage polarization), and signaling pathways (e.g., NF-κB, MAPK, *GBP2*-*OTUD5*-*GPX4*) (6, 7), with exosome regulation, ferroptosis, pyroptosis (8), and mitochondrial autophagy (9) emerging as novel regulatory mechanisms and potential drug targets. Nevertheless, SALI is a multifactorial and multilevel condition. There is limited evidence on the interactions among immune cells, the regulation of multi-signaling pathway networks, and clinical translation (7, 10, 11). Metabolic reprogramming plays a crucial role in immune regulation (12–14). Recent studies (15, 16) have indicated that metabolites can affect disease progression by modulating genes and cellular functions. The regulatory mechanism of “metabolites–key genes–immune cells” in SALI remains elusive, and there is a lack of systematic research at the multi-omics level.

Xuanbai Chengqi Decoction (XCD) is composed primarily of raw gypsum, raw rhubarb, bitter apricot kernel, and *Trichosanthes kirilowii* peel. Preliminary UHPLC-QQQ analysis detected relatively high levels of amygdalin, rhein, aloe-emodin, rutin, and diosmetin in the administered XCD; these compounds were therefore considered major candidate constituents rather than confirmed active ingredients. XCD is a classic formula in clinical treatment for lung heat with interior fullness and lung injury (17), widely used in severe conditions such as sepsis, COPD, and respiratory failure (18–20). Preclinical studies have reported its anti-inflammatory, immunomodulatory, pulmonary- and intestinal-protective effects (19, 21). In ALI, the efficacy of XCD in alleviating lung damage has been demonstrated through experimental methods such as *in vivo* studies (17, 19, 20). However, the key metabolic targets, core regulatory genes, and immunological mechanisms involved in XCD-mediated intervention of SALI remain unclear.

In light of the aforementioned research background and existing issues, this study centers on sepsis-associated acute lung injury as the research subject. Through the integration of metabolomics data from animal models, public transcriptomics, and single-cell sequencing, and in combination with multiple bioinformatics analysis approaches, key serum metabolites and core regulatory genes during the disease progression were screened and prioritized. The intrinsic correlation between key molecules and immune cells (T cells) is clarified, and the metabolic-gene-immune interactive regulatory network is systematically analyzed. The objective is to more precisely elucidate the molecular pathogenesis of sepsis-associated acute lung injury and offer a scientific foundation and novel perspectives for the early identification and potential intervention of the disease.

## 2 Materials and methods

### 2.1 Ethics

All animal work was performed in accordance with the Guidelines for the Review of Laboratory Animal Ethics and the ARRIVE 2.0 recommendations (https://arriveguidelines.org). The protocol was approved by the Animal Ethics Committee of the Second Affiliated Hospital of Liaoning University of Traditional Chinese Medicine (Ethics Approval No.: LZYY250412). Animal housing, experimental procedures, and euthanasia followed ARRIVE 2.0 principles to safeguard welfare and minimize avoidable suffering.

### 2.2 ALI animal model

Thirty male Wistar rats (180–220 g) were acclimated for 5 days and then randomly assigned to the control, LPS (5 mg/kg body weight), or XCD-treated (10.8 g/kg) group (n = 10 per group). Food was withdrawn on the evening of day 3. On day 4, all animals except those in the blank control group were anesthetized, placed supine, and administered LPS intratracheally under laryngoscopic visualization. The assigned oral regimens were resumed after recovery. Intratracheal LPS was chosen because it directly targets the lung and produces a sepsis-like systemic inflammatory response with predominant pulmonary injury, which corresponds to the core clinical phenotype of SALI; this model has been widely used in SALI/ALI research. However, it differs from cecal ligation and puncture (CLP) in that it uses a single agonist rather than polymicrobial sepsis and does not reproduce the full systemic multi-organ involvement of CLP. On day 7, corresponding to 72 hours after LPS administration and consistent with the acute phase of SALI, blood, alveolar lavage fluid, and tissue specimens were collected under propofol anesthesia (22). Euthanasia was performed by injecting propofol emulsion (60mg/kg) via the tail vein, after which blood was collected from the abdominal aorta, lung tissue was harvested post-mortem.

### 2.3 Validation of the ALI model

Model validation included determination of the lung wet-to-dry (W/D) ratio and hematoxylin-eosin (HE) histology. Concentrations of TNF-α, IL-10, IFN-γ, IL-6, and IL-1β were measured by multiplex secretome analysis (Cat. No. XMPlex03240735, XMplex Rat Cytokine 5-Plex Panel) according to the manufacturer’s instructions, with technical assistance from SXM Biotechnology Co., Ltd. (Wuhan, China). Statistical processing was completed in GraphPad Prism 10 and SPSS 27.0. Results were presented as bar charts or as the mean ± standard error. Group differences were tested by one-way analysis of variance followed by LSD multiple comparisons, and *P* < 0.05 was considered statistically significant (23, 24).

### 2.4 Untargeted metabolomics analysis

Untargeted serum profiling was performed by liquid chromatography-tandem mass spectrometry (LC-MS/MS) for the control, LPS (ALI model), and XCD groups. Samples stored at −80 °C were thawed and processed on ice. After vortexing for 10 s, 50 μL of serum was mixed with 300 μL of internal-standard extraction solution containing 20% acetonitrile in methanol and vortexed for 3 min. The mixture was centrifuged at 12,000 r/min for 10 min at 4 °C. Next, 200 μL of supernatant was retained at −20 °C for 30 min and centrifuged again at 12,000 r/min for 3 min at 4 °C. A final 180-μL aliquot of supernatant was transferred to an autosampler-vial insert for instrumental analysis.

Chromatographic separation was carried out on a Vanquish ultra-high-performance liquid chromatography system (Thermo Scientific, Massachusetts, USA) equipped with a Waters ACQUITY Premier HSS T3 column (1.8 µm, 2.1 mm × 100 mm). Mobile phase A was aqueous 0.1% formic acid, whereas mobile phase B was acetonitrile containing 0.1% formic acid. The column temperature, flow rate, and injection volume were 40 °C, 0.4 mL/min, and 4 μL, respectively. The gradient elution program was as follows: 0–1 min, B phase linearly increased from 5% to 20%; 1–3 min, B phase linearly increased from 20% to 99%; 3–4.5 min, maintained at 99% B; 4.5–4.6 min, B phase decreased from 99% to 5%; and 4.6–6.0 min, equilibrated at 5% B.

Data acquisition alternated full-scan MS with data-dependent MS/MS under dynamic-exclusion conditions. Electrospray ionization was operated in positive and negative modes across m/z 75–1000 at a resolving power of 35,000. The remaining parameters were set as follows: spray voltage, 3.5 kV in positive mode and 3.2 kV in negative mode; sheath gas, 30 Arb; auxiliary gas, 5; ion-transfer-tube temperature, 320 °C; vaporizer temperature, 300 °C; collision energies, 30, 40, and 50 V; signal-intensity threshold, 1.00E + 06 cps; Top N/Top speed, 10; and exclusion duration, 3 s.

### 2.5 Metabolomics data analysis

Raw mass-spectrometry files were converted to mzML with ProteoWizard before peak detection, alignment, and retention-time correction in XCMS. Features with a missing rate exceeding 50% within each experimental group were excluded from further analysis. For the remaining features, missing values were imputed using a combined KNN and one-fifth-minimum strategy: values with a missing proportion greater than 50% were replaced with one-fifth of the minimum detected value for that feature (a conservative imputation approach), whereas values with a missing proportion of 50% or less were estimated using KNN imputation, thereby preserving the quantitative structure of the metabolomic dataset while minimizing the introduction of bias. Support vector regression (SVR) was subsequently applied to normalize peak areas.

For annotation, the normalized and filtered features were queried against an internal laboratory library, integrated public resources, and prediction databases. Assignments were retained only when the overall identification score exceeded 0.5 and the coefficient of variation (CV) in quality-control (QC) samples was below 0.3. Positive- and negative-ion records were consolidated by retaining the entry with the highest identification confidence and lowest CV, thereby generating the final metabolite matrix.

Global sample variation was evaluated by principal component analysis (PCA) using the “pca” function in mixOmics (v 6.26.0) (25), with separate contrasts for control versus LPS and LPS versus XCD. Partial least-squares discriminant analysis (PLS-DA) was then performed with “plsda” to examine group separation along the principal components in both comparisons. Orthogonal PLS-DA using the “opls” function provided an additional assessment of the models’ ability to distinguish the groups.

Differential metabolites (DEMs) were initially prioritized using nominal P < 0.05, VIP > 1.5, and |log_2_FC| > 0 as an exploratory screening strategy. Because |log_2_FC| > 0 alone does not represent a biologically meaningful effect-size threshold, we additionally performed Benjamini–Hochberg (BH) correction and sensitivity analyses across |log_2_FC| thresholds (0, 0.05, 0.10, 0.20, 0.30, 0.50, and 1.00). The BH and sensitivity results are reported in the Results and **Supplementary Table 1**, **Supplementary Figure 4** (26). To justify the use of a permissive |log_2_FC| > 0 threshold and confirm data reliability, we assessed technical variability by calculating the coefficient of variation (CV = standard deviation/mean) for all metabolites within each experimental group. CV distributions were visualized using boxplots and cumulative distribution function (CDF) plots generated with ggplot2 (v 3.5.1) (27), and were evaluated against the commonly accepted acceptability threshold of CV < 0.3. DEM distributions were visualized as volcano plots with ggplot2 (v 3.5.1), whereas ComplexHeatmap (v 2.21.1) (28) displayed the five largest increases and five largest decreases ranked by |log_2_FC|. Metabolites that changed in opposite directions across the two contrasts were intersected with ggvenn (v 0.1.10; https://CRAN.R-project/package=ggvenn). The overlapping set was defined as candidate metabolites associated with XCD intervention in acute lung injury (ALI).

### 2.6 Collection of public data and transcriptome analysis

The bulk-expression datasets GSE66890 (GPL6244) and GSE32707 (GPL10558), together with the single-cell dataset GSE151263 (GPL20301), were downloaded from GEO (https://www.ncbi.nlm.nih.gov/geo/). GSE66890 was used as the training cohort and contained whole-blood samples from 29 patients with sepsis complicated by ARDS (SALI group) and 28 patients with sepsis alone (sepsis group) (29). The validation cohort, GSE32707, comprised 31 sepsis + ARDS and 58 sepsis-only whole-blood samples (30). GSE151263 included PBMC samples from 3 patients with sepsis-related ARDS and 4 patients with sepsis alone. All datasets were accessed on February 6, 2026. Candidate metabolites with corresponding records in the IEU OpenGWAS repository (https://opengwas.io/datasets/) were designated as key metabolites, and the relevant GWAS summary statistics were obtained.

In the training cohort, differentially expressed genes (DEGs) between SALI and sepsis were detected with limma (v 3.56.2) (31) using |log_2_FC| > log2(1.3), approximately 0.3785 (32), and *P* < 0.05. The overall DEG distribution was depicted with ggplot2. ComplexHeatmap was used to display expression patterns for the 10 most upregulated and 10 most downregulated genes ranked by |log_2_FC|. For cross-species validation, the rat LPS-induced ALI dataset GSE158832 (GPL18694) and the mouse CLP-induced sepsis-ALI dataset GSE165226 were additionally obtained from GEO.

### 2.7 Single-cell RNA sequencing (scRNA-seq) data analysis

GSE151263 was processed with Seurat (v 5.3.0) (33). Quality control retained cells meeting 200 < nFeature_RNA < 2,500, nCount_RNA < 15,000, and a mitochondrial-transcript proportion below 10%. Harmony was subsequently used to integrate samples and reduce batch-associated variation. NormalizeData performed normalization, and FindVariableFeatures with the vst option ranked genes according to cell-to-cell variability; the top 2,000 highly variable genes (HVGs) were selected for subsequent analyses.

ScaleData was applied to the normalized matrix, after which RunPCA calculated principal components from the HVGs. Statistically supported components were evaluated with JackStrawPlot (*P* < 0.05), and ElbowPlot was inspected to assess the variance structure. Unsupervised clustering was performed with FindNeighbors and FindClusters, and RunUMAP projected the resulting clusters into two dimensions.

Cell clusters were annotated using marker genes supported by the literature (34). The proportion of each cell type was then calculated and visualized separately for the SALI and sepsis groups.

### 2.8 Single-cell phenotype-associated genome-GWAS analysis

For every candidate metabolite represented by GWAS data, that metabolite was defined as the phenotype. Single-cell Polygenic Architecture-driven GWAS integration was performed with scPagwas (v 2.0.0) (35) and TwoSampleMR (v 0.6.14) (36) to relate phenotype-associated genetic signals to the annotated cell populations. Bootstrap resampling of the GWAS summary statistics assessed the stability of cell-specific genetic-signal contributions (*P* < 0.05). The cell population with the strongest association was prioritized for downstream analyses. The specific parameters for scPagwas were set as follows: minor allele frequency (MAF) threshold = 0.5, single-nucleotide polymorphism (SNP) significance P-value = 5×10^−8^, bootstrap iterations = 10, singlecell = TRUE, celltype = TRUE, and n.cores = 1 to enforce single-threaded execution and avoid parallel environment conflicts.

### 2.9 High-dimensional weighted gene co-expression network analysis (hdWGCNA)

High-dimensional weighted gene co-expression network analysis was conducted in GSE151263 to identify modules relevant to the biology of the prioritized cell type in SALI. Sample-specific metacells were generated with MetacellsByGroups in hdWGCNA (v 0.4.05) (37). TestSoftPowers was used to screen soft-thresholding power values with a predefined scale-free topology criterion of scale-free R² ≥ 0.80 and mean connectivity < 200, and ConstructNetwork then built the weighted network. TOM-based hierarchical clustering was performed with hierarchicalCluster using a minimum module size of 50 and a module-merging threshold of 0.2; Pearson correlations among modules were also visualized. Modules with comparatively high expression in the prioritized cell type were retained, and all genes within those modules were defined as target module genes. Other key parameters: genes expressed in at least 5 % of cells were retained, k-nearest neighbors = 25, max_shared = 10, grouping performed by cell type and sample origin. Module expression scores were calculated via the UCell method based on top 25 hub genes within each module.

### 2.10 Enrichment and interaction analysis of candidate genes

Candidate genes were obtained by intersecting the target module genes with bulk DEGs in ggvenn, thereby linking the resulting set to SALI, the candidate metabolite, and the prioritized cell population. Gene Ontology and Kyoto Encyclopedia of Genes and Genomes enrichment analyses were conducted with org.Hs.eg.db (v 3.18.0) and clusterProfiler (v 4.8.3) (38), with *P* < 0.05 used to identify enriched terms. Protein-protein interactions were retrieved from STRING (http://string-db.org) at a minimum confidence score of 0.15, and the network was visualized with circlize (v 0.4.16) (39).

### 2.11 Machine learning and SHapley Additive exPlanations (SHAP) interpretation

Candidate-gene prioritization in the training cohort used a machine-learning workflow. Before model fitting, recursive feature elimination in caret with 5-fold cross-validation was applied to remove redundant predictors and reduce overfitting. Ten classifiers were examined: logistic regression, random forest, decision tree, bagging tree, stochastic gradient boosting trees, linear discriminant analysis, support vector machine, neural network, naive Bayes, and k-nearest neighbor. Performance was summarized using AUC, sensitivity, specificity, accuracy, F1 score, positive predictive value, negative predictive value, and Brier score. For each classifier, pROC (v 1.19.0.1) (40) generated ROC curves for the training and test subsets produced by 5-fold cross-validation, and models satisfying 1 > AUC > 0.7 were considered. To further evaluate the calibration and clinical utility of the selected model, calibration curve and decision curve analyses (DCA) were performed on the training set. For the calibration curve, predicted probabilities were binned into 10 equal intervals and plotted against observed proportions, with a LOESS-smoothed curve overlaid against the ideal diagonal. DCA was conducted using 50 bootstrap resamples to calculate net clinical benefit across threshold probabilities, comparing the model against “treat all” and “treat none” strategies. The model with the most favorable overall metric profile was selected, and its retained features were treated as candidate key genes. SHAP values calculated with fastshap (v 0.1.1) were used for model interpretation.

### 2.12 Expression validation and gene set enrichment analysis (GSEA)

Candidate key-gene expression was compared between SALI and sepsis with the Wilcoxon test (*P* < 0.05). A gene was defined as a key gene only when both the training and validation cohorts showed a statistically significant change in the same direction. Its SALI-associated functions were then evaluated by gene set enrichment analysis (GSEA) in the training cohort, using the MSigDB C5 collection of GO biological processes as the reference.

For each key gene, psych (v 2.4.3) (41) was used to calculate Spearman correlations between its expression and all other genes in the training dataset. Genes were ranked from the highest to the lowest coefficient and submitted to clusterProfiler. Significant enrichment required |normalized enrichment score (NES)| > 1 and adjusted P value (p.adjust) < 0.05. Biological-process terms shared among the key genes were summarized in an intersection plot generated with ggvenn.

### 2.13 Cross-species validation

To evaluate the cross-species reproducibility of the candidate genes, two independent public animal transcriptomic datasets were obtained from the Gene Expression Omnibus (GEO). The rat LPS-induced acute lung injury dataset GSE158832 (GPL18694), generated using the Illumina HiSeq 2500 platform, included three control lung samples and three LPS-induced ALI lung samples. The mouse sepsis-associated acute lung injury dataset GSE165226 included six control lung samples and six cecal ligation and puncture (CLP)-induced sepsis-ALI lung samples.

The rat and mouse orthologs corresponding to the human candidate genes GIMAP4, RCBTB2, and WBP2 were identified in the respective expression matrices. Differential expression analysis was performed separately in each dataset using the limma package. For each candidate gene, log2 fold change and P values were calculated, and P values for the three candidate genes within each dataset were adjusted using the Benjamini–Hochberg method. Cross-species consistency was assessed according to the direction of expression change and statistical significance across the human, rat, and mouse datasets.

### 2.14 Immune infiltration and molecular docking

Immune infiltration across 28 cell populations was quantified in the training dataset by ssGSEA implemented in GSVA (v 1.50.5) (42). The Wilcoxon test compared infiltration scores between SALI and sepsis (*P* < 0.05). Spearman correlations between key genes and immune populations that differed between groups were calculated and visualized with corrplot (v 0.92) (43); statistical significance required *P* < 0.05 and |cor| > 0.3.

Candidate-metabolite structures were downloaded from PubChem (https://pubchem.ncbi.nlm.nih.gov/) in SDF format, whereas PDB-format structures of proteins encoded by the key genes were retrieved from UniProt (https://www.uniprot.org/). Molecular docking was performed with CB-Dock2 (https://badd.labshare.cn/cb-dock2/php/index.php). Predicted ligand-protein complexes with binding energy ≤ −5.0 kcal/mol were considered computationally favorable in-silico predictions. Such outputs do not constitute proof of physical binding and require biophysical assays such as SPR or ITC together with mutational experiments for verification.

### 2.15 Surface plasmon resonance

Prepare Immobilization running buffer: 1.0× PBS-P+ (pH 7.4); Interaction (kinetics) running buffer: 1.0× PBS-P+ (pH 7.4) with 1% (v/v) DMSO. Load CM5 sensor chip, use 1.0× PBS-P+ as running buffer,Channel activation: Inject 1:1 (v/v) 0.4 M EDC/0.1 M NHS mixture into active Channel 2 at 10 μL/min for 420 s to activate chip surface carboxymethyl dextran groups. Ligand coupling: Dilute ligand protein to 50 μg/mL in 10 mM sodium acetate buffer, inject at 10 μL/min for 420 s for covalent binding to activated chip surface. Channel blocking: Inject 1 M ethanolamine hydrochloride (pH 8.5) at 10 μL/min for 420 s to block residual unreacted active ester groups. Reference channel: Follow same activation/blocking procedures, inject protein-free 10 mM sodium acetate buffer during coupling at same flow rate and duration, as background subtraction control to eliminate effects of non-specific binding and bulk refractive index changes. Dilute small molecule compounds for kinetic analysis in 96-well microplate with PBST to an initial concentration of 25,600 nM, then serially dilute down to 25 nM. Analyze all concentration gradients, set association time to 100 s and dissociation time to 120 s. Regenerate the sensor chip with 10 mM glycine-HCl (pH 2.0) for 5 minutes after each flow cycle, repeat until all measurements are completed.

### 2.16 Cellular functional analysis

Single-cell gene set variation analysis (scGSVA) scored the MSigDB C2:CP: KEGG collection across the annotated cell populations. Independently, KEGG metabolic-pathway activity was estimated with the AUCell method in scMetabolism (v 0.2.1) (44). Gene-regulatory-network activity was inferred in parallel with dorothea (v 1.12.0) (45), enabling identification of transcription factors with high activity across cell types.

### 2.17 Cell-cell communication and pseudotime analysis

CellChat (v 1.6.1) (46) was used to infer intercellular signaling, including the number and aggregate strength of communications and the ligand-receptor pairs that supported them. Cells from the prioritized population were re-embedded and re-clustered using resolution = 0.1 and dims = 10, followed by subtype annotation (47, 48). Monocle (v 2.30.1) (49) reconstructed cell trajectories and pseudotime and was used to evaluate changes in key-gene expression along the inferred progression.

### 2.18 scTenifoldKnk analysis

Potential consequences of key-gene loss were simulated in the prioritized cells with scTenifoldKnk. A gene-regulatory network was reconstructed after each virtual knockdown, and genes with altered inferred regulation were selected at |Z| > 1 and *P* < 0.05. These genes were overlapped with the SALI DEGs to obtain disease-associated transcripts potentially perturbed by knockdown.

### 2.19 Statistical analysis

All analyses were performed in R v 4.3.1. Unless otherwise stated, the Wilcoxon test was used for between-group comparisons, with *P* < 0.05 indicating statistical significance. Correlations were calculated using psych (v 2.4.3) and were considered significant when *P* < 0.05 and |cor| > 0.3.

## 3 Results

### 3.1 XCD significantly alleviates pulmonary injury in the SALI rat model

Model establishment and the response to XCD were assessed using lung histopathology, body-weight trajectories, lung wet-to-dry ratios, and circulating TNF-α, IL-10, IFN-γ, IL-6, and IL-1β. Alveolar architecture remained orderly in controls, whereas LPS produced alveolar congestion and inflammatory-cell infiltration; both lesions were visibly reduced after XCD treatment (**Figure 1A**). Control animals gained weight throughout the observation period. In contrast, the trajectory in the LPS group differed significantly from that in controls (*P* < 0.01): weight increased on days 1-3, changed little on day 4 because of preoperative fasting and water restriction, and decreased on days 5-7. XCD treatment attenuated the loss observed during days 5–7 relative to the LPS group (**Figure 1C**). The lung W/D ratio was higher after LPS than in controls (*P* < 0.01) and was lower after XCD than after LPS (*P* < 0.01) (**Figure 1B**). Each of the five cytokines increased in the LPS group versus controls (*P* < 0.05) and decreased significantly following XCD treatment (*P* < 0.05) (**Figures 1E–I**).

**Figure 1:**
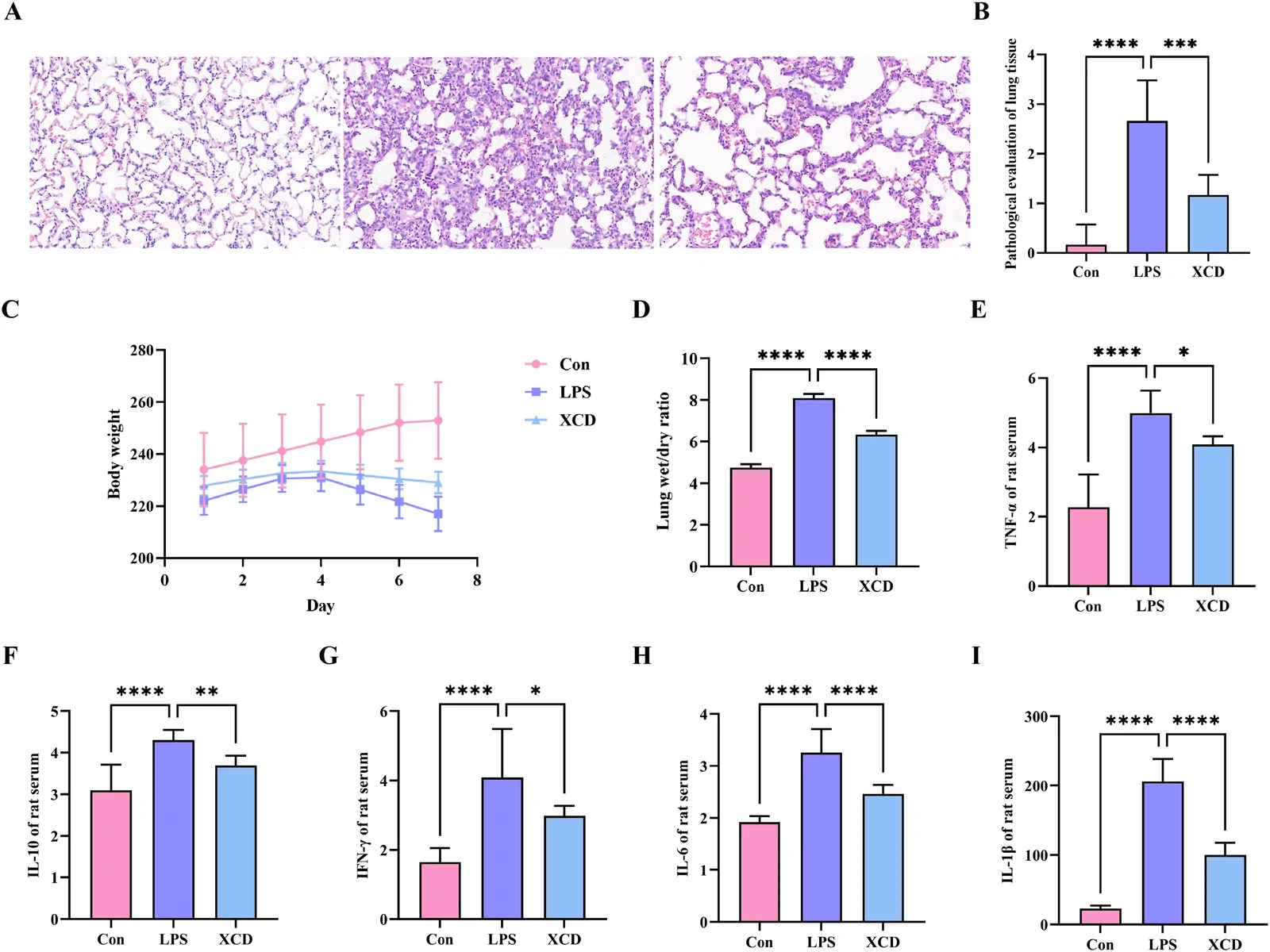
Xuanbai Chengqi Decoction alleviates lipopolysaccharide-induced lung injury in rats. **(A)** Representative hematoxylin-eosin-stained lung sections from the control, LPS, and XCD groups. **(B)** Pathological evaluation of lung tissue. **(C)** Body-weight trajectories during the experimental period. **(D)** Lung wet-to-dry weight ratio.**(E**–**I)** Serum concentrations of TNF-α, IL-6, IFN-γ, IL-1β, and IL-10, respectively. Data are presented as the mean ± standard error. *P < 0.05, **P < 0.01, and ****P < 0.0001.

### 3.2 Screening of 34 candidate metabolites for XCD intervention in SALI

Untargeted analysis detected 1,472 metabolites (**Supplementary Table 1**). In the PCA plots, the LPS group separated from controls, and the XCD group separated from the LPS group along the first two components (**Supplementary Figures 1A, B**). PLS-DA and OPLS-DA produced similar discrimination for both comparisons (**Supplementary Figures 1C–F**): the paired groups diverged along P1, and their 95% confidence ellipses did not overlap. These multivariate patterns indicated broad serum-metabolome changes after LPS exposure and following XCD treatment and supported clear distinction of the comparison groups. To further evaluate data reliability and technical variability, we calculated the intra-group CV for all metabolites in each experimental group. In the LPS vs Con comparison, 100% of metabolites in both the Con and LPS groups had CV < 0.3, with median CV values of 0.019 and 0.021, respectively. In the LPS vs XCD comparison, all metabolites in both the LPS and XCD groups also had CV < 0.3 (100%), with median CV values of 0.021 and 0.020, respectively (**Supplementary Figures 1G, H**; **Supplementary Table 1**). These results demonstrate exceptionally low intra-group measurement variability and high data quality, supporting that the DEMs identified under the |log_2_FC| > 0 threshold are unlikely to be dominated by technical noise.

Relative to controls, LPS altered 179 metabolites, of which 74 increased and 105 decreased (**Figure 2A**). Comparing XCD with LPS identified 61 DEMs, including 34 higher and 27 lower metabolites after treatment (**Figure 2B**). Intersecting changes that occurred in opposite directions across the two contrasts yielded 34 metabolites potentially related to XCD intervention in SALI (**Figure 2C**; **Supplementary Table 2**). After BH correction, no metabolite in the LPS vs. Control comparison reached FDR < 0.05, whereas 132 metabolites in the XCD vs. LPS comparison reached FDR < 0.05. Sensitivity analysis showed that the number of opposite-direction candidates decreased to 5 at |log_2_FC| > 0.05 and to 0 at |log_2_FC| > 0.10. These findings indicate that the metabolite changes are predominantly small in effect size, and the candidate list should be regarded as exploratory. Only Carnitine C18:1 (oleoylcarnitine) corresponded to an IEU OpenGWAS record (met-a-681). This European-ancestry GWAS comprised 7,708 participants and 2,545,666 SNPs.

**Figure 2:**
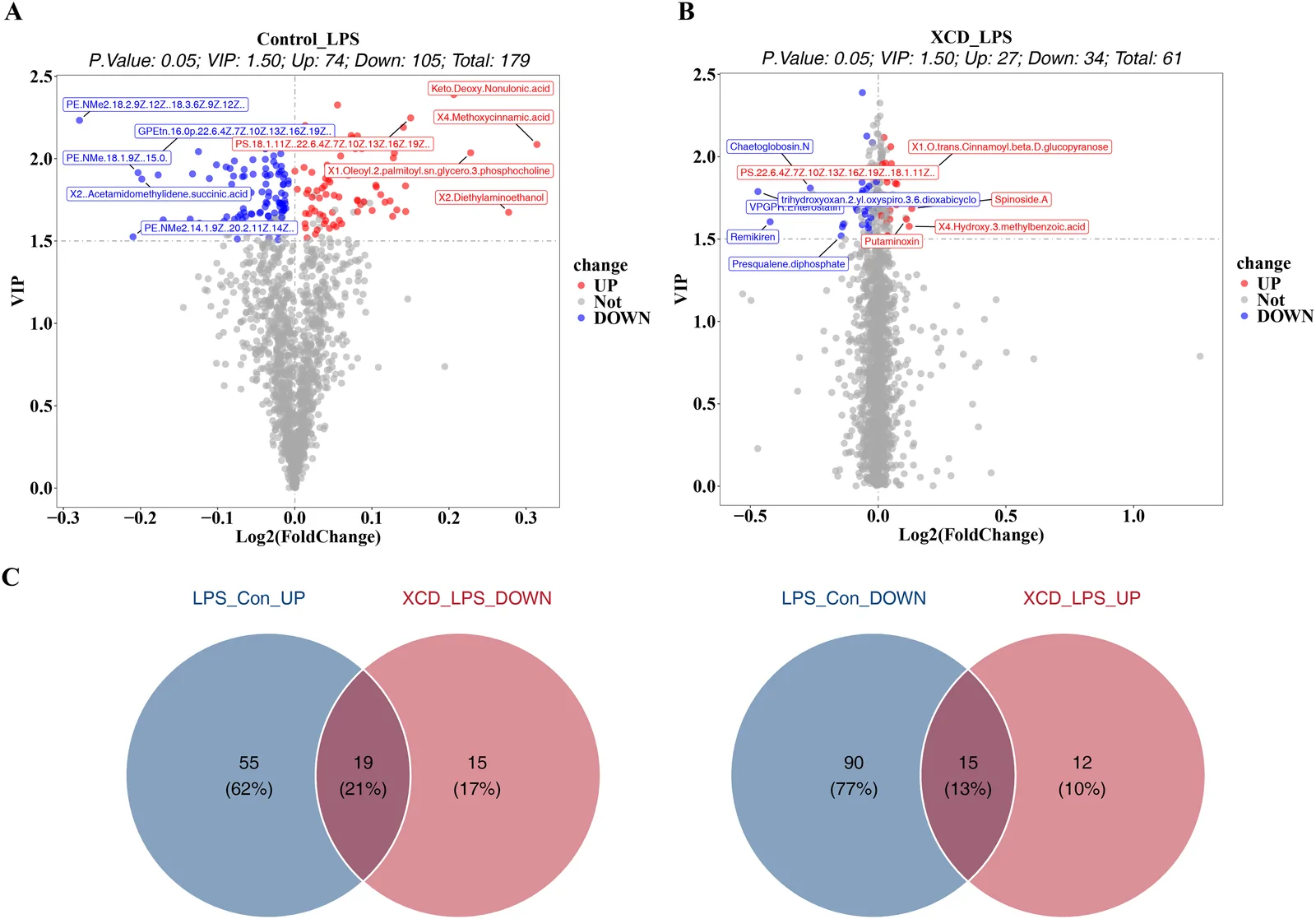
Differential serum metabolites associated with XCD intervention. **(A)** Volcano plot of metabolites in the LPS group versus the control group. **(B)** Volcano plot of metabolites in the XCD group versus the LPS group. **(C)** Intersections between metabolites increased by LPS and decreased by XCD and metabolites decreased by LPS and increased by XCD.

### 3.3 Stable genetic association between oleoylcarnitine and T cells

Quality control retained 24,245 cells expressing 16,790 genes (**Supplementary Figures 2A, B**). The 30 principal components that contributed most strongly to variation among the top 2,000 HVGs were used for clustering (*P* < 0.001; **Supplementary Figures 2C–E**). Unsupervised analysis resolved 11 clusters (**Figure 3A**), which were annotated into seven major lineages using reported markers: CD14+ monocytes (*S100A9*, *S100A8*, *S100A12*, *VCAN*, *FCN1*, *LYZ*, and *MNDA*), T cells (*CD3D*, *CD3E*, *CD3G*, and *TRAC*), B cells (*CD79A*, *CD79B*, *MS4A1*, and *CD19*), NK cells (*NKG7*, *GNLY*, *KLRD1*, and *GZMB*), CD16+ monocytes (*LST1*, *IFITM3*, *MS4A7*, *AIF1*, *CDKN1C*, *SAT1*, *FCGR3A*, and *PPBP*), megakaryocytes (*PPBP*, *PF4*, *NRGN*, *GNG11*, and *TUBB1*), and dendritic cells (*PPP1R14B*, *UGCG*, *PLD4*, *HLA-DQA1*, and *FCER1A*) (**Figures 3B, C**). CD14+ monocytes and T cells were the most abundant populations in both groups (**Figure 3D**).

**Figure 3:**
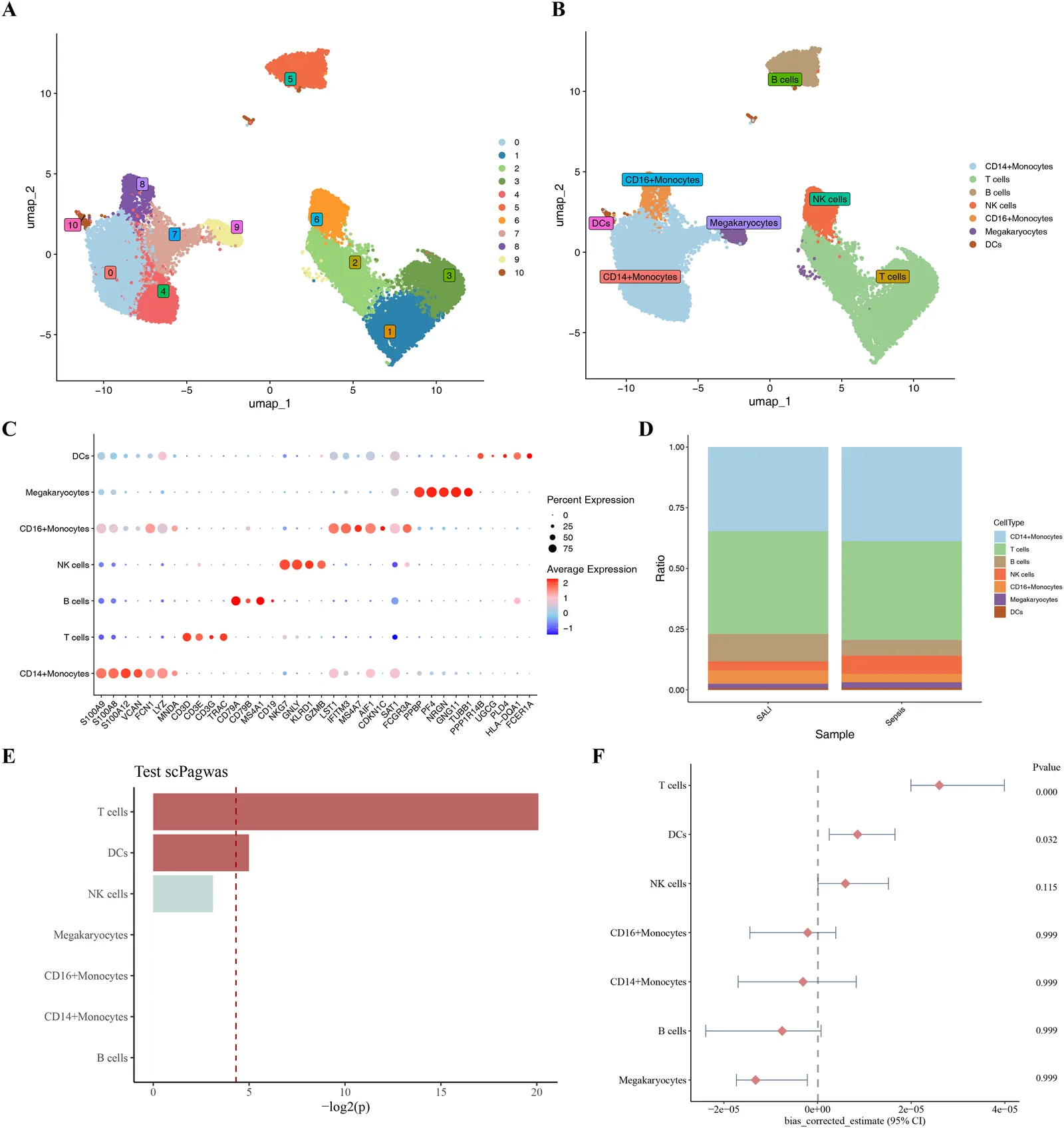
Single-cell landscape and cell-specific genetic association with oleoylcarnitine. **(A)** UMAP visualization of 11 cell clusters. **(B)** Annotation of seven major cell populations. **(C)** Dot plot of canonical marker-gene expression. **(D)** Relative cell-type proportions in the sepsis and SALI groups. **(E)** scPagwas-based cell-type association with oleoylcarnitine-related genetic signals. **(F)** Bootstrap estimates and 95% confidence intervals for cell-specific associations.

Cell-specific genetic-signal analysis identified T cells showing the strongest positive genetic correlation with the oleoylcarnitine phenotype (P < 0.001; 95% confidence interval > 0). This positive correlation was stable across bootstrap resampling, demonstrating a statistical association between T-cell genetic architecture and oleoylcarnitine-related polygenic signals rather than proving causal regulation. Accordingly, T cells were selected as the prioritized cell population for downstream correlative analyses (**Figures 3E, F**).

### 3.4 Identification of 1,567 T-cell signature genes

A soft-thresholding power of 7 produced an approximately scale-free topology (R² = 0.815) (**Figure 4A**). TOM-based clustering divided the network into five modules (**Figure 4B**). The turquoise module correlated positively with the yellow and brown modules, whereas the blue and yellow modules showed a strong negative correlation (**Figure 4C**). Because genes in the turquoise and blue modules exhibited comparatively high activity in T cells, the two modules were combined to define 1,567 T-cell signature genes (**Figures 4D, E**; **Supplementary Table 3**).

**Figure 4:**
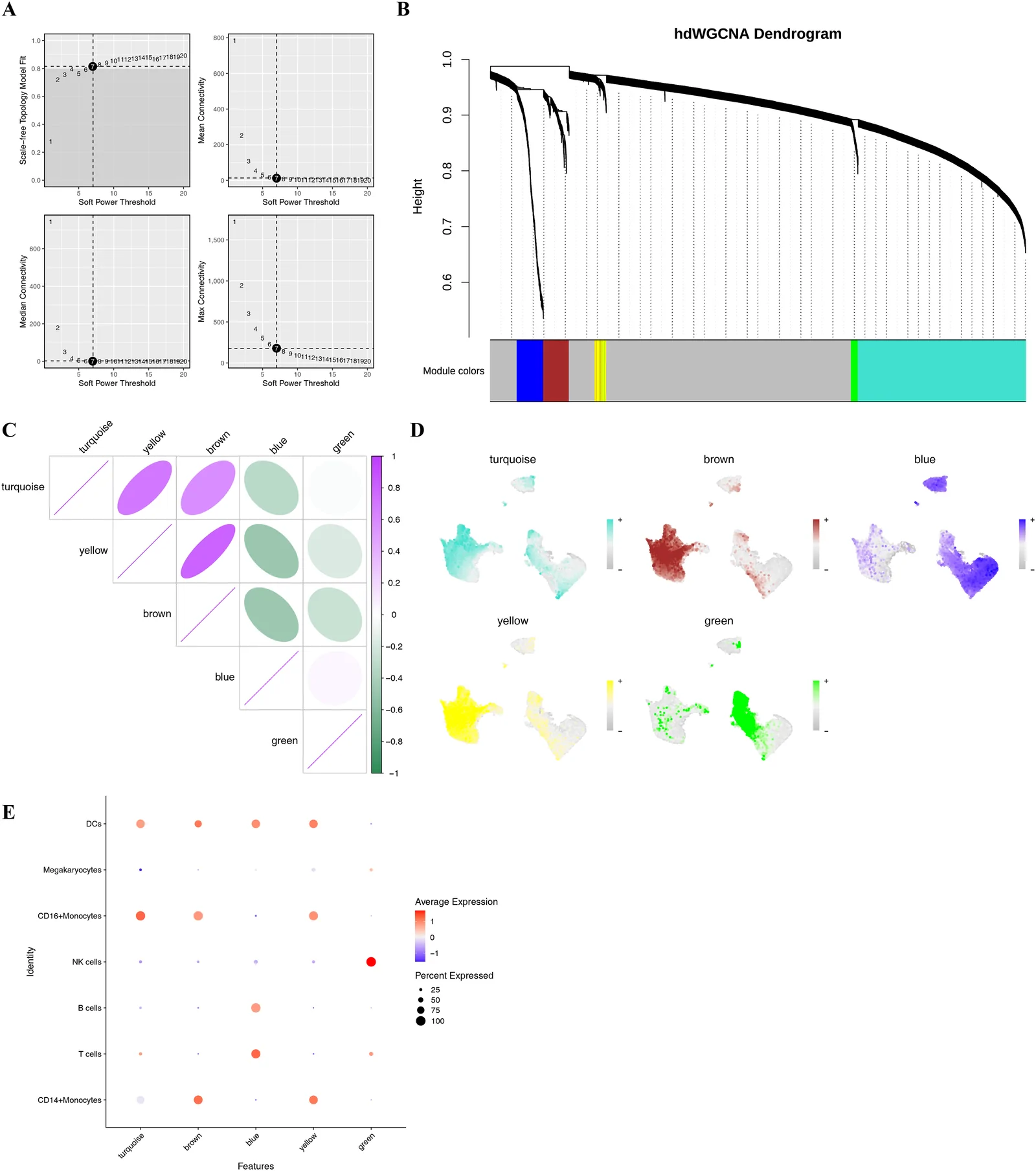
Identification of T-cell co-expression modules by hdWGCNA. **(A)** Selection of the soft-thresholding power. **(B)** Hierarchical clustering dendrogram and module assignments. **(C)** Correlations among module eigengenes. **(D)** UMAP visualization of module-gene activity across cell populations. **(E)** Relative module expression across the annotated cell types.

### 3.5 Identification of 20 candidate genes and the functional and interaction analyses

Using the training cohort GSE66890, we identified 271 SALI-versus-sepsis DEGs, including 151 upregulated and 120 downregulated genes (**Figures 5A, B**). Overlap of these DEGs with the 1,567 T-cell signature genes identified 20 candidates (**Figure 5C**). Thus, the candidate set simultaneously reflected SALI-associated expression differences, T-cell co-expression structure, and the cell phenotype linked to oleoylcarnitine.

**Figure 5:**
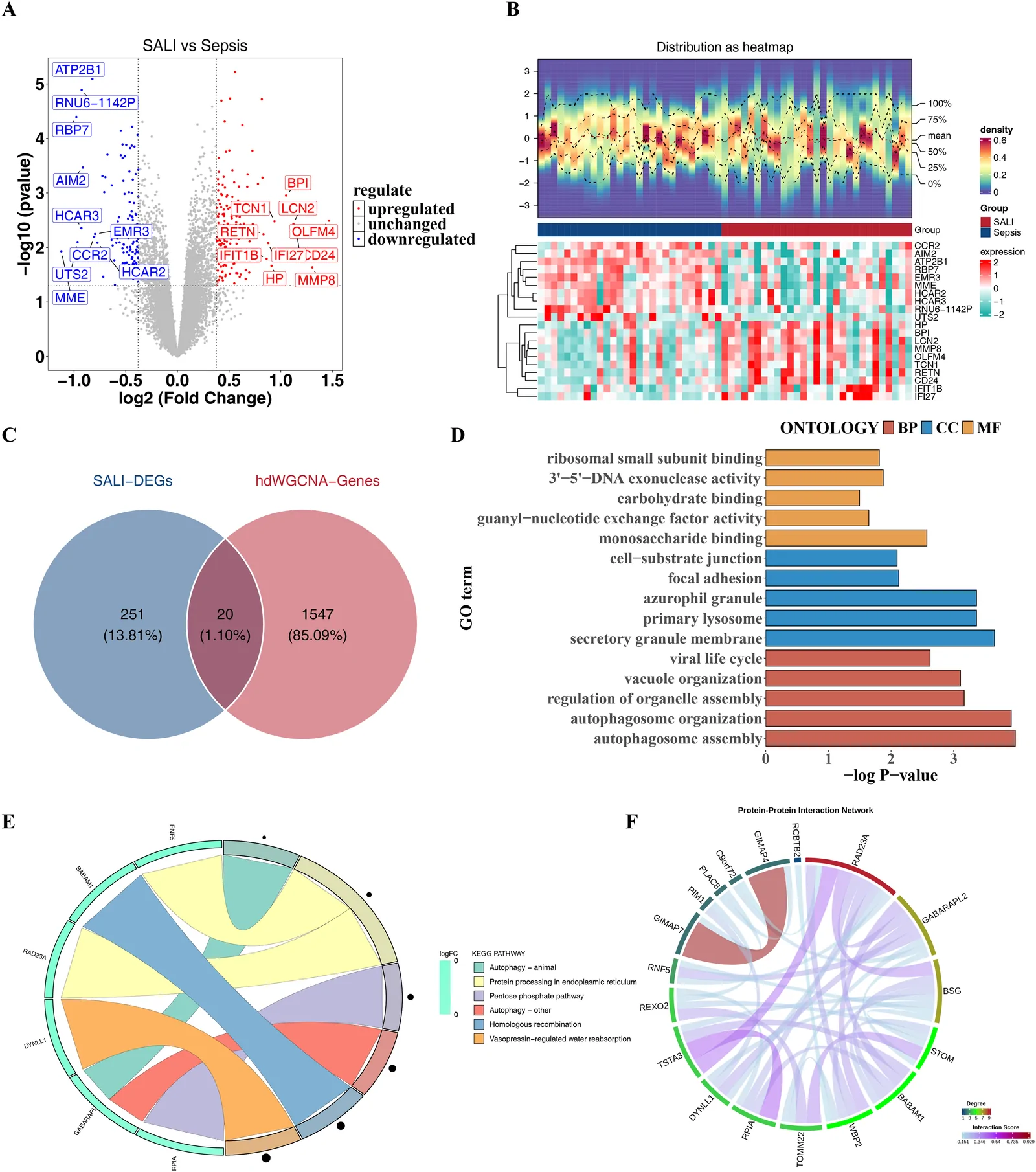
Identification and functional characterization of candidate genes. **(A)** Volcano plot of SALI-versus-sepsis differentially expressed genes. **(B)** Heatmap of the top upregulated and downregulated genes. **(C)** Overlap between differentially expressed genes and T-cell signature genes. **(D)** Representative Gene Ontology enrichment terms. **(E)** Enriched Kyoto Encyclopedia of Genes and Genomes pathways. **(F)** Protein-protein interaction network of the candidate genes.

Functional enrichment of the 20 candidates returned 132 biological-process terms, including “ribosomal small subunit binding” and “3’-5’-DNA exonuclease activity”; 36 cellular-component terms, including “cell-substrate junction” and “focal adhesion”; and 24 molecular-function terms, including “viral life cycle” and “vacuole organization” (**Figure 5D**; **Supplementary Table 4**). Six KEGG pathways were also enriched, including “protein processing in endoplasmic reticulum” (**Figure 5E**).

Pairwise connections were present among 18 candidate genes in the interaction network. *GIMAP7*-*GIMAP4* showed the highest interaction score, and *RAD23A* was linked to nine candidates (**Figure 5F**).

### 3.6 The NB machine-learning model was selected as the optimal model

Based on the training cohort GSE66890, RFE first evaluated the stability of the 10 highest-ranked candidates across the 10 algorithms (**Supplementary Figure 3A**). *C9orf72* ranked first in BT, KNN, LDA, NNET, RF, SGBT, and SVM; *REXO2* was the leading feature in LR and NB; and *PIM1* ranked first in DT. Considering the complete performance profile, NB provided the most balanced results, with AUC = 0.788, sensitivity = 0.753, accuracy = 0.560, F1 score = 0.683, PPV = 0.647, and NPV = 0.674 (**Supplementary Figure 3B**). Because the NB AUC exceeded 0.7 in both the training and test subsets, this model was selected as optimal. Its retained features were *C9orf72*, *GIMAP4*, *PIM1*, *GLIPR1*, *RNF5*, *REXO2*, *GABARAPL2*, *SYNE2*, *WBP2*, *DYNLL1*, *RCBTB2*, *STOM*, and *PLAC8* (**Figures 6A, B**). Calibration curve analysis demonstrated good calibration, with the LOESS-smoothed curve deviating minimally from the ideal diagonal. Decision curve analysis further showed that the NB model yielded higher net clinical benefit than both the “treat all” and “treat none” strategies across a wide range of threshold probabilities, suggesting its potential auxiliary value in clinical decision-making (**Supplementary Figures 3C, D**).

**Figure 6:**
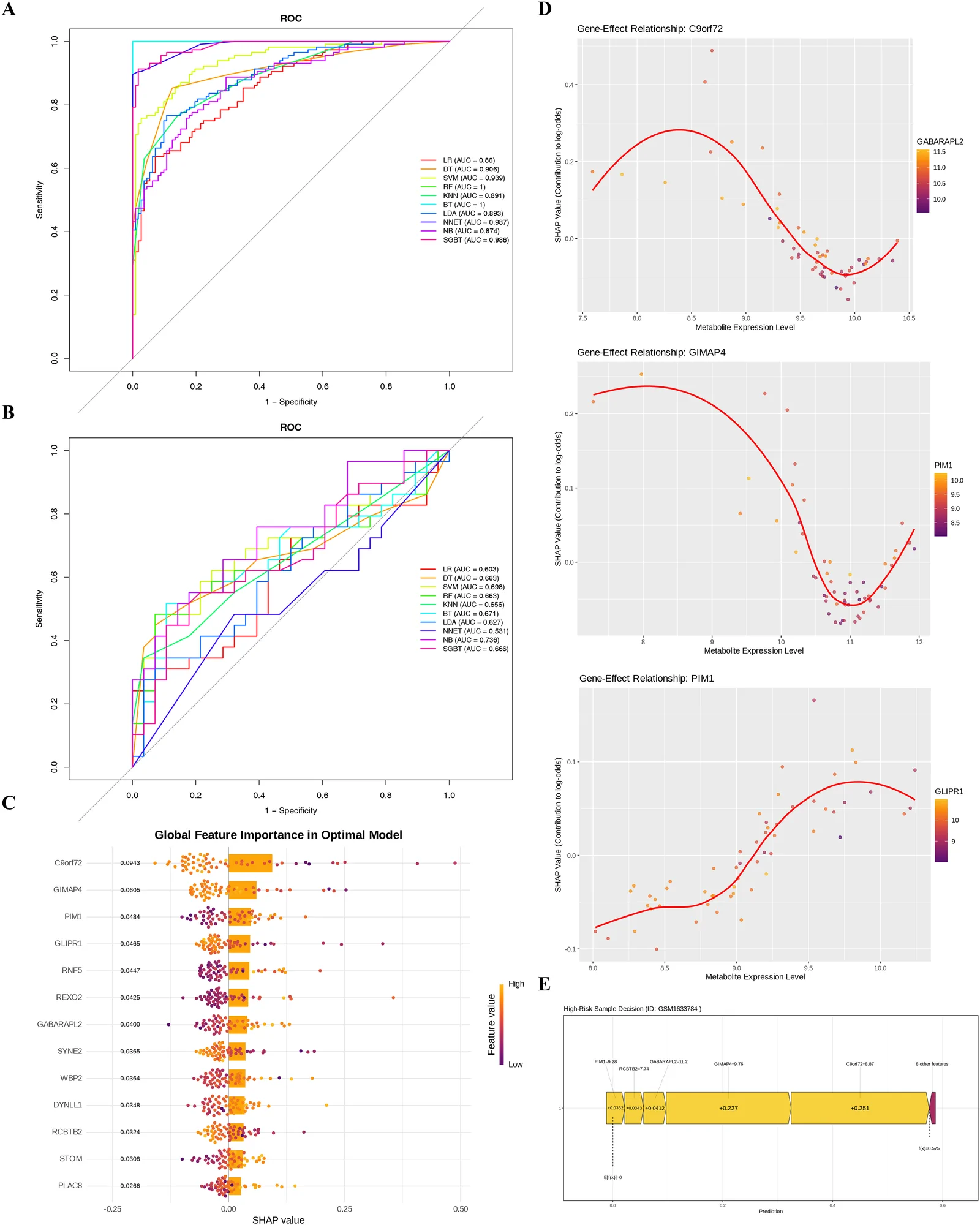
Machine-learning selection and interpretation of candidate hub genes. **(A)** Receiver operating characteristic curves for the 10 classifiers in the training subsets. **(B)** Receiver operating characteristic curves in the test subsets. **(C)** SHAP summary of feature importance in the selected naive Bayes model. **(D)** SHAP dependence plots for *C9orf72*, *GIMAP4*, and *PIM1*. **(E)** Individual SHAP explanation for sample GSM1633784.

SHAP analysis identified *C9orf72*, *GIMAP4*, and *PIM1* as the strongest contributors to NB predictions, with nonlinear relationships between expression and model output. Lower expression of *C9orf72* and *GIMAP4* shifted predictions toward SALI, as did higher *PIM1* expression (**Figures 6C, D**). GSM1633784 was randomly chosen for an individual-level explanation. Its cumulative feature contribution was 0.575, assigning the sample to the high-risk class (**Figure 6E**). Every displayed feature increased the prediction relative to the baseline value of 0, and no negative contribution was observed, indicating a profile strongly classified as SALI by the model.

### 3.7 *GIMAP4*, *RCBTB2* were prioritized as candidate hub genes, and *WBP2* was listed as candidate gene requiring validation

In both the (GSE66890) and validation cohort (GSE32707), *GIMAP4* and *RCBTB2* were expressed at lower levels in SALI than in sepsis (*P* < 0.05). *WBP2* expression was higher in SALI, but the difference did not reach significance (*P* > 0.05) (**Figures 7A, B**). GIMAP4 and RCBTB2 therefore received concordant statistical support from expression validation in both cohorts. Given its contribution to the machine-learning model and functional enrichment with the other two genes, WBP2 was retained as a candidate gene requiring further validation, rather than as a prioritized hub gene.

**Figure 7:**
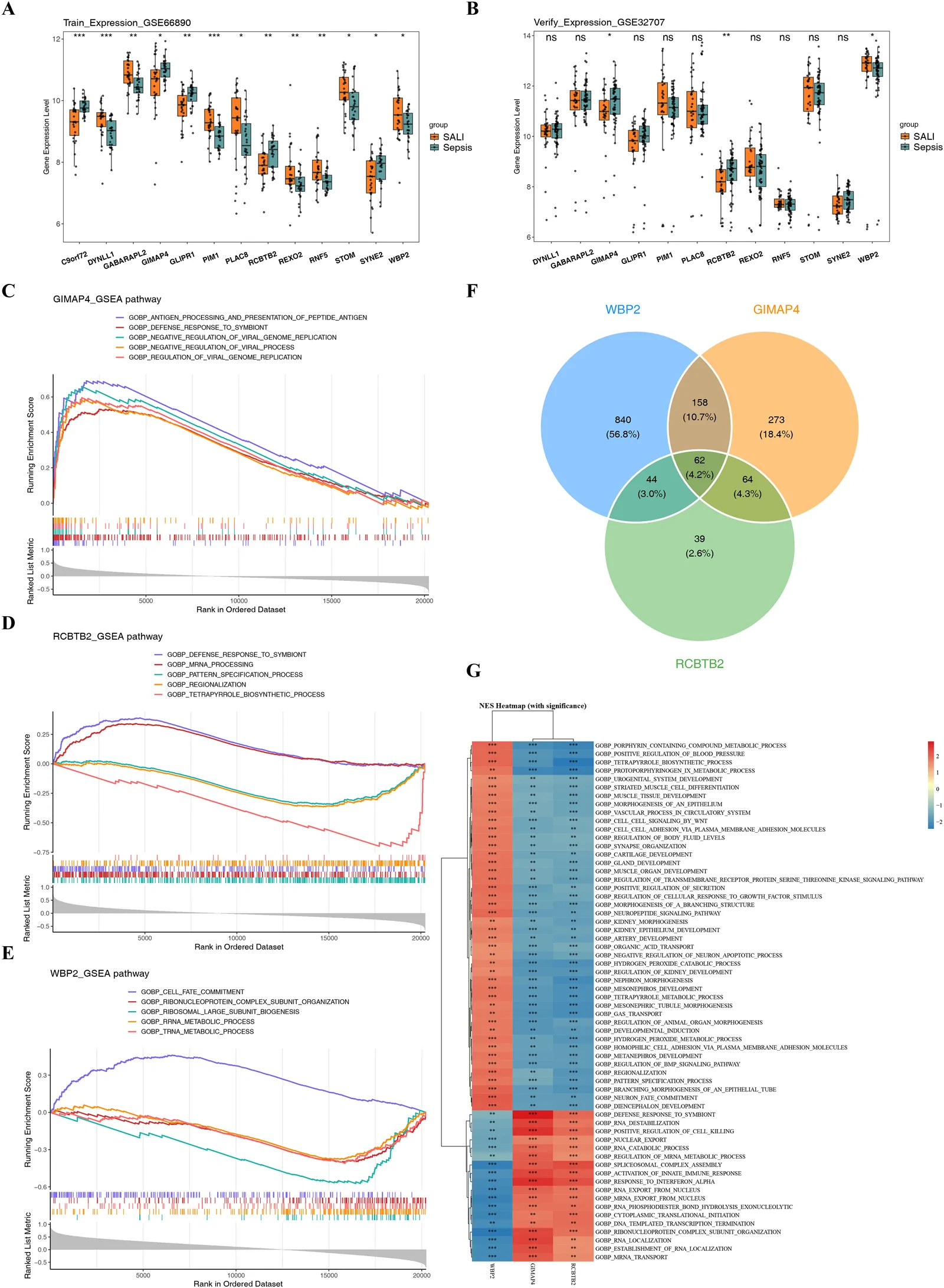
Expression validation and gene set enrichment analysis of candidate hub genes and candidate gene requiring validation. **(A, B)** Expression of GIMAP4, RCBTB2, and WBP2 in the training and validation cohorts. Note that WBP2 did not reach statistical significance in the validation cohort and is therefore designated as a candidate requiring further validation. **(C–E)** Representative enriched biological processes for *GIMAP4*, *RCBTB2*, and *WBP2*, respectively. **(F)** Overlap of enriched biological processes among the three genes. **(G)** Heatmap of the 62 shared biological.

Training-set GSEA associated *GIMAP4* with 557 biological processes; among the five terms with the smallest adjusted P values was “antigen processing and presentation of peptide antigen” (**Figure 7C**; **Supplementary Table 5**). *RCBTB2* was linked to 209 processes, including “defense response to symbiont” (**Figure 7D**; **Supplementary Table 6**). *WBP2* was associated with 1,104 processes, with “cell fate commitment” included among the representative terms (**Figure 7E**; **Supplementary Table 7**).

Intersection of the three enrichment profiles yielded 62 shared biological processes, including “ribonucleoprotein complex subunit organization” and “regionalization” (**Figures 7F, G**). This overlap suggests that the candidate hub genes and candidate gene requiring validation are associated with related biological programs in SALI, but it does not establish functional interdependence among them.

To evaluate cross-species consistency, we analyzed two independent animal datasets. In rat LPS-ALI (GSE158832), GIMAP4 was significantly downregulated (log_2_FC = −0.614, *P* = 2.64 × 10^−4^, BH-adjusted *P* = 7.93 × 10^−4^), whereas RCBTB2 and WBP2 were not significantly changed. In mouse CLP-ALI (GSE165226), GIMAP4 (log_2_FC = −0.575, *P* = 0.00378, BH-adjusted *P* = 0.0113) and RCBTB2 (log_2_FC = −0.230, *P* = 0.0138, BH-adjusted *P* = 0.0207) were significantly downregulated, whereas WBP2 showed no significant change (log_2_FC = 0.0011, *P* = 0.991). Thus, GIMAP4 showed consistent downregulation across human, rat, and mouse; RCBTB2 showed human–mouse consistency; and WBP2 lacked stable cross-species support. For details of the analysis results, please refer to **Supplementary Tables 2**-**4**, **Supplementary Figures 5**, **6**.

### 3.8 Candidate hub genes are associated with immune-cell infiltration and predicted oleoylcarnitine binding

Using the training cohort GSE66890, type 2 T-helper-cell scores were zero in all samples. Among the remaining populations, CD56bright natural killer cells were more abundant in SALI than in sepsis (*P* < 0.05), whereas eosinophils, macrophages, myeloid-derived suppressor cells, natural killer T cells, and neutrophils were less abundant (*P* < 0.05) (**Figures 8A, B**). The candidate hub genes were correlated with these differentially represented populations. In particular, eosinophil scores showed a positive correlation with *RCBTB2* (cor = 0.646, *P* < 0.001) and a negative correlation with *WBP2* (cor = -0.479, *P* < 0.001) (**Figure 8C**).

**Figure 8:**
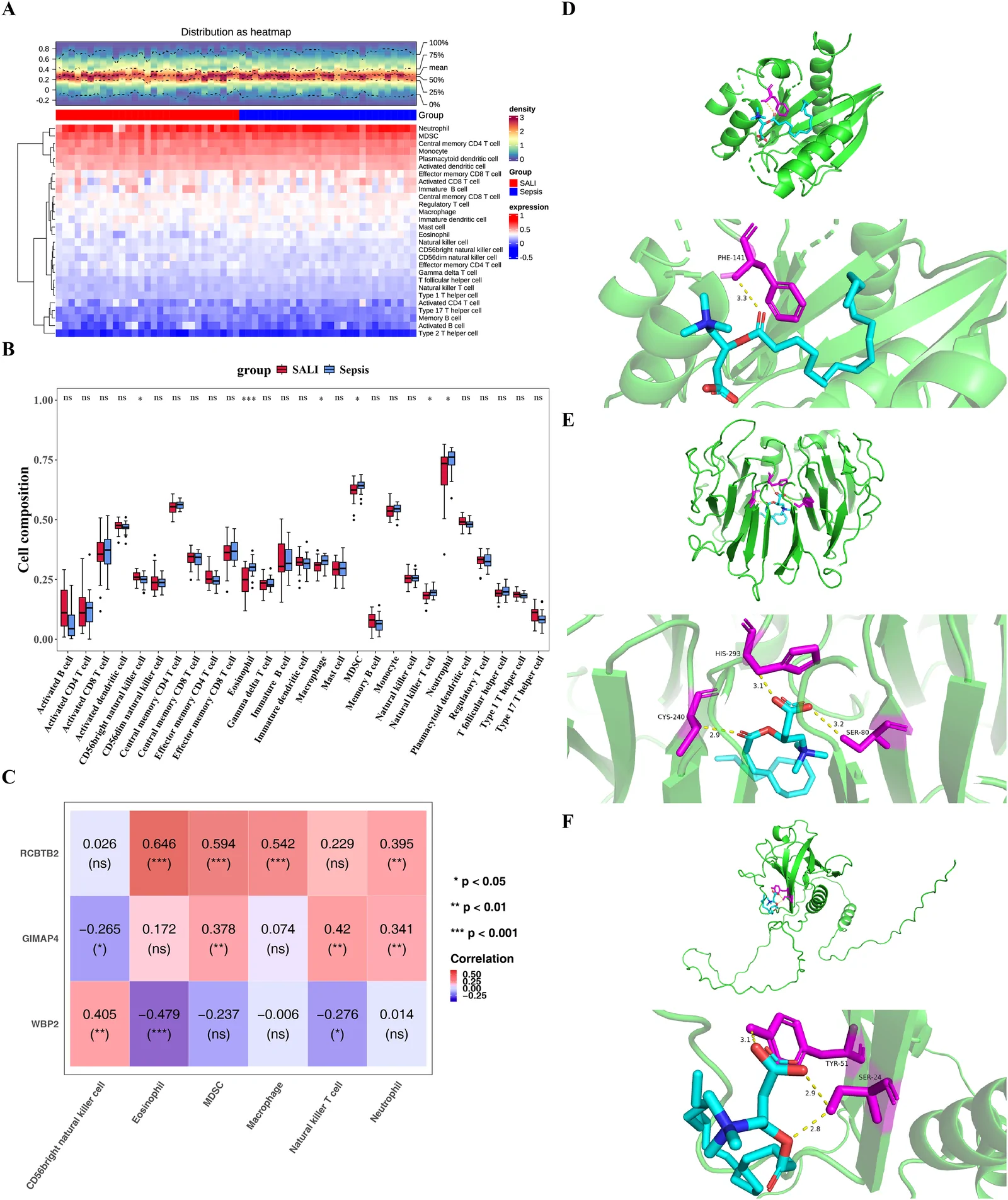
Immune infiltration and molecular docking. **(A, B)** Comparison of immune-cell infiltration scores between the sepsis and SALI groups. **(C)** Correlations between candidate hub genes and differentially represented immune-cell populations. **(D–F)** Predicted docking conformations and binding pockets of oleoylcarnitine with GIMAP4, RCBTB2, and WBP2, respectively.

**Table 1** summarizes the docking results. The predicted GIMAP4-oleoylcarnitine complex had an energy of -5.6 kcal/mol and involved PHE-141 (**Figure 8D**). Docking to RCBTB2 yielded an energy of -6.0 kcal/mol, with HIS-293, CYS-240, and SER-80 at the modeled interface (**Figure 8E**). For WBP2, the predicted energy was -4.4 kcal/mol, and TYR-51 and SER-24 were involved in the modeled complex (**Figure 8F**).

**Table 1:** Binding energy between key genes and oleoylcarnitine.

Gene	ID	Ligand	CID	Binding energy (kcal/mol)
GIMAP4	3LXX	Carnitine.C18.1	CID6441392	-5.6
RCBTB2	AF-O95199-2-F1	Carnitine.C18.1	CID6441392	-6.0
WBP2	AF-Q969T9-F1	Carnitine.C18.1	CID6441392	-4.4

The table summarizes molecular docking of the three candidate genes with oleoylcarnitine (Carnitine.C18.1; PubChem CID: 6441392). ID, protein structure identifier.

### 3.9 Oleoylcarnitine binds directly to RCBTB2 and GIMAP4

Surface Plasmon Resonance (SPR) results demonstrated that, across the concentration range of 25–25600 nM, the binding affinity of oleoylcarnitine for both RCBTB2 and GIMAP4 exhibited a concentration-dependent behavior, accompanied by a relatively fast dissociation rate. Kinetic and fitting analyses revealed the equilibrium dissociation constants (KD) for oleoylcarnitine and RCBTB2, GIMAP4 to be 1.08×10^−5^ M and 4.16×10^−5^ M (**Figures 9A, B**), steady-state fitting analyses revealed the steady-state KD for oleoylcarnitine and RCBTB2, GIMAP4 to be 10.1 μM and 10.4 μM (**Figures 9C, D**), respectively-both values falling within the micromolar range, consistent with moderate binding strength.

**Figure 9:**
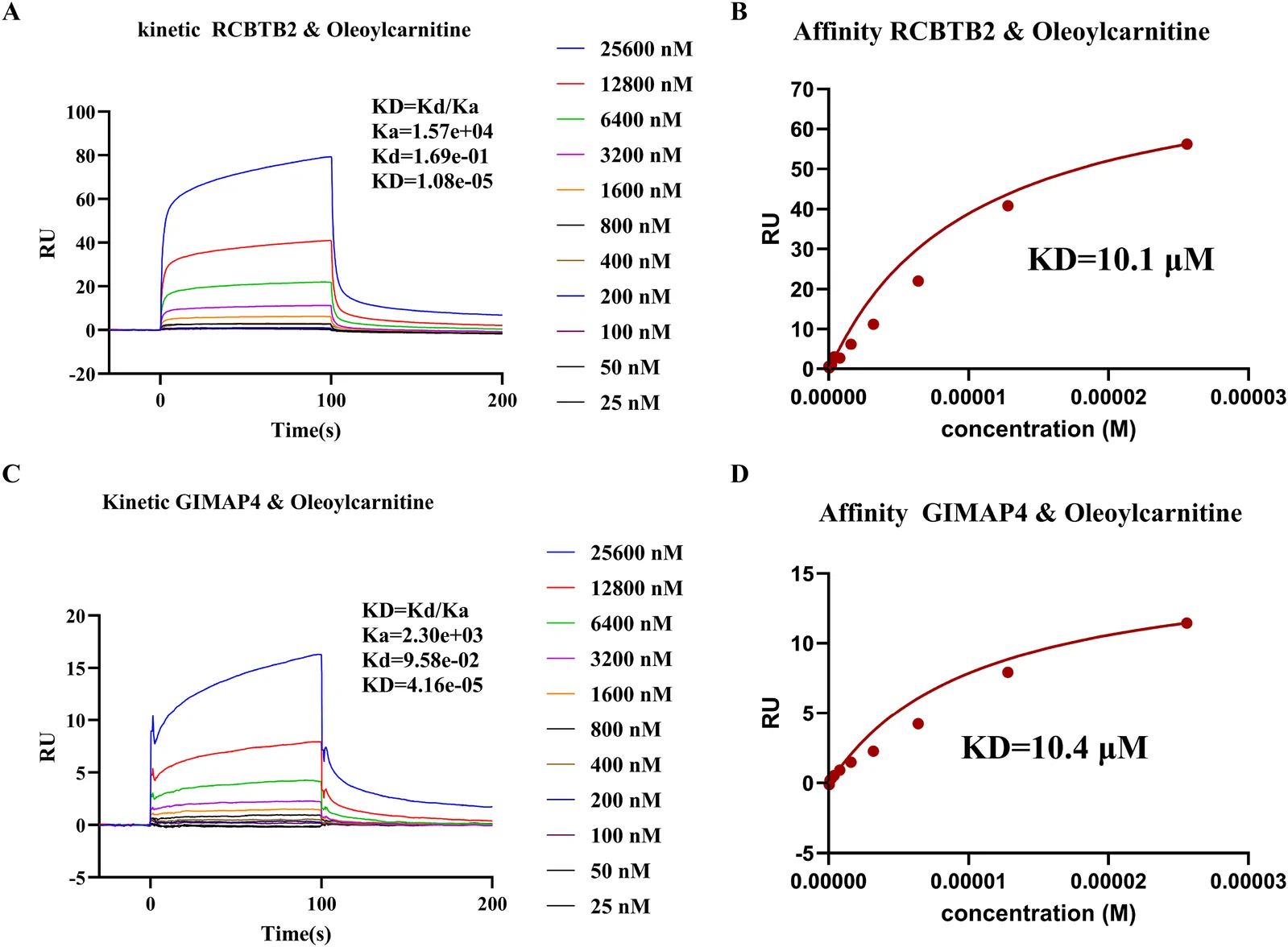
Surface Plasmon Resonance. **(A)** Protein RCBTB2 and Oleoylcarnitine kinetic binding curve plot. **(B)** Protein RCBTB2 and Oleoylcarnitine steady-state binding curve plot. **(C)** Protein GIMAP4 and Oleoylcarnitine kinetic binding curve plot. **(D)** Protein GIMAP4 and Oleoylcarnitine steady-state binding curve plot.

### 3.10 Metabolic pathways are activated in T cells with characteristic transcriptional regulatory patterns

Single-cell pathway scoring indicated higher activity of alanine, aspartate and glutamate metabolism and aminoacyl-tRNA biosynthesis in T cells, together with lower activity of the acute myeloid leukemia and adherens junction gene sets (**Figure 10A**). Citrate-cycle and pyruvate-metabolism activity was also increased in T cells (**Figure 10B**). Collectively, these patterns indicate a distinct metabolic state and greater biosynthetic activity in the SALI T-cell compartment.

**Figure 10:**
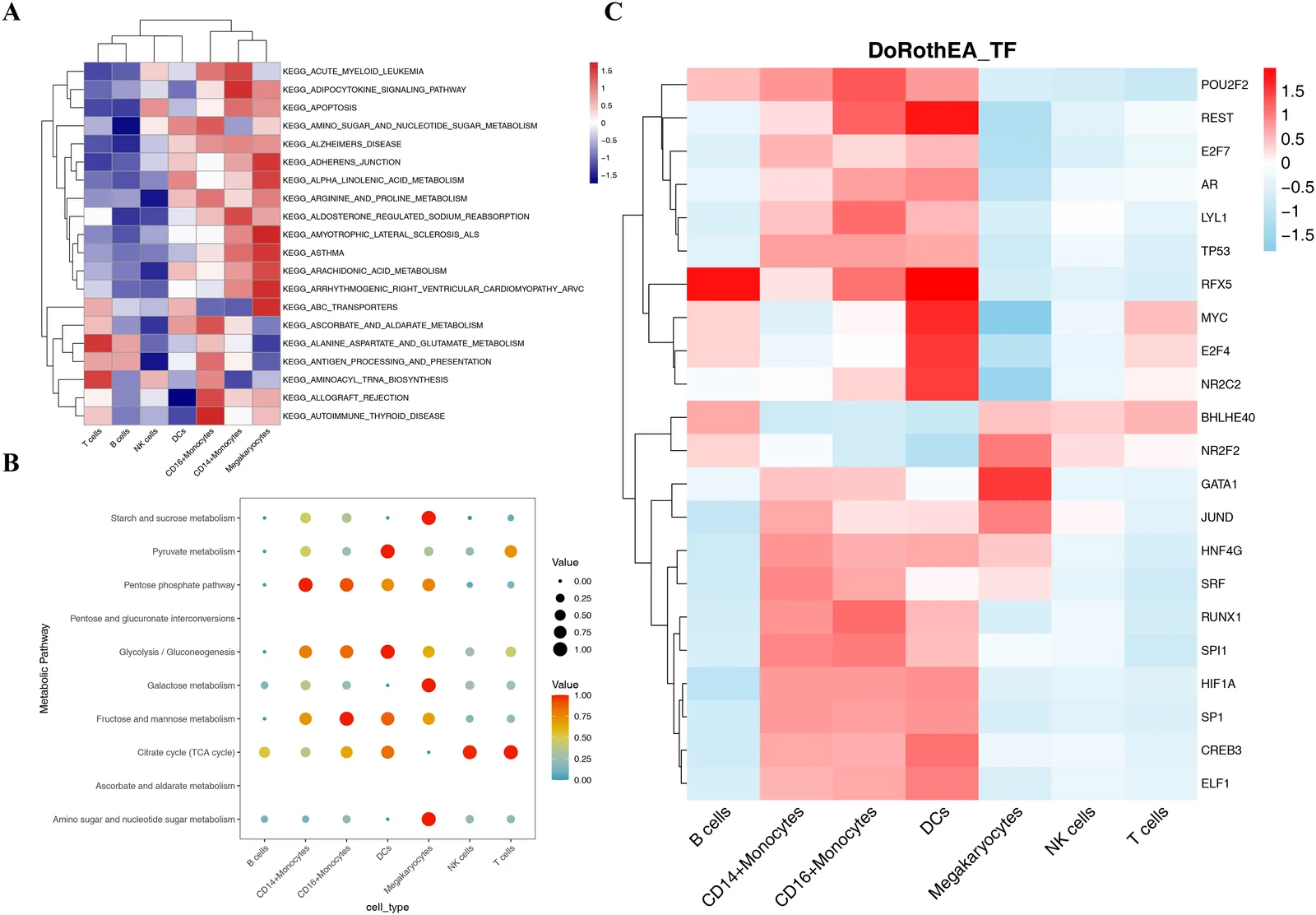
Functional and regulatory states across single-cell populations. **(A)** Relative activity of representative KEGG pathways estimated by single-cell gene set variation analysis. **(B)** Metabolic-pathway activity estimated with scMetabolism. **(C)** Transcription-factor activity inferred with DoRothEA.

Transcription-factor inference showed high RFX5, REST, and related activities in dendritic cells and high GATA1 activity in megakaryocytes. MYC and BHLHE40 displayed intermediate activity in T cells (**Figure 10C**).

### 3.11 Enhanced cellular communication in SALI

Both the estimated number of communications and their aggregate strength were greater in SALI than in sepsis. The largest increases in interaction count involved CD14+ monocytes communicating with CD16+ monocytes, B cells, and NK cells, together with T cells communicating with CD16+ monocytes (**Figure 11A**). Communication strength between T cells and CD14+ monocytes was also higher in SALI (**Figure 11B**).

**Figure 11:**
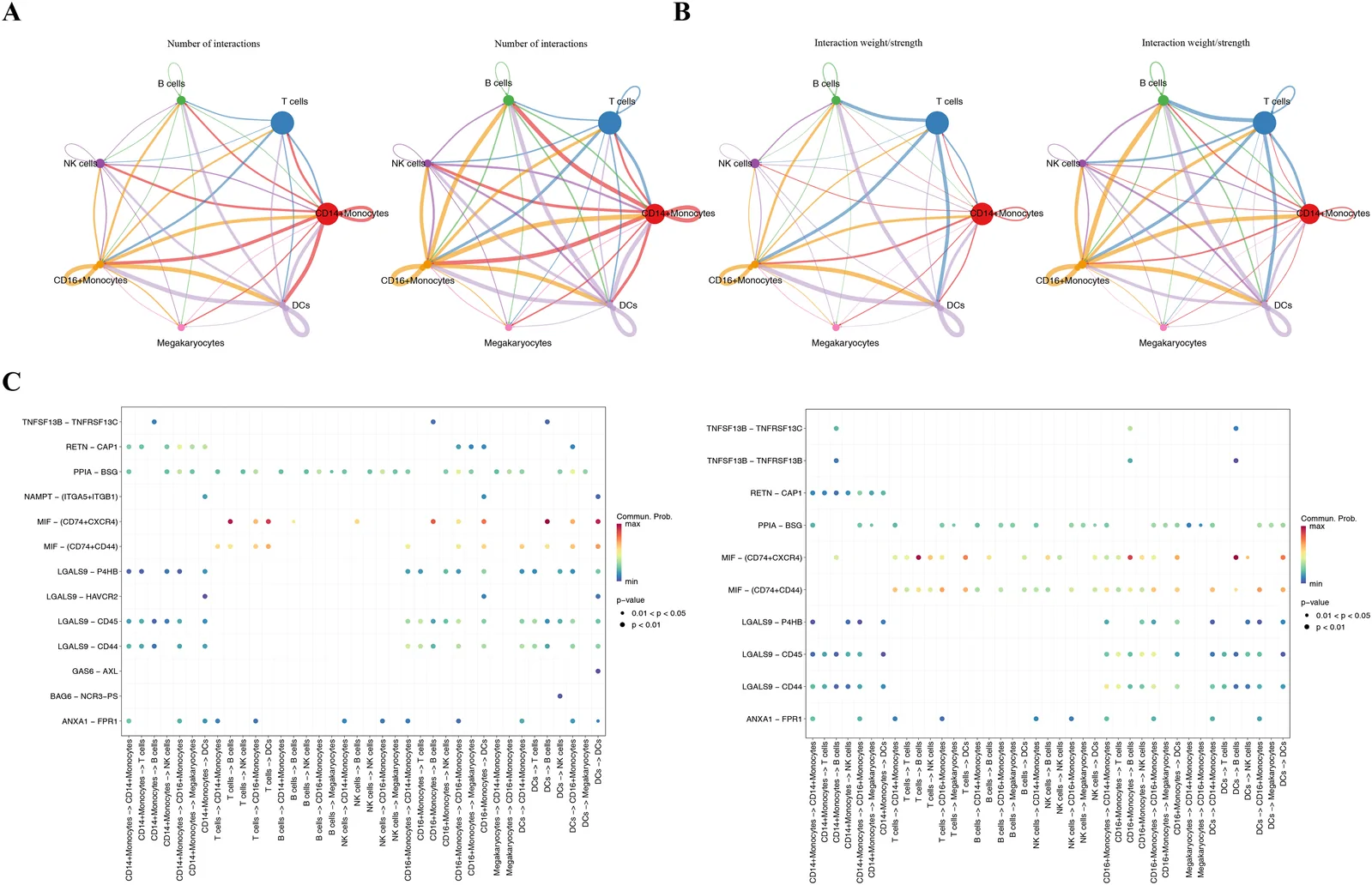
Remodeling of cell-cell communication in SALI. **(A)** Inferred numbers of intercellular interactions in the sepsis and SALI groups. **(B)** Aggregate interaction strengths in the two groups. **(C)** Ligand-receptor signaling patterns, including the MIF-(CD74+CXCR4) and MIF-(CD74+CD44) axes.

### 3.12 Dynamic effects of candidate hub genes and candidate gene requiring validation in T cells

T cells functioned as signal senders in both groups, mainly through the inferred MIF-(CD74+CXCR4) and MIF-(CD74+CD44) axes. Compared with sepsis, SALI showed stronger T-cell-to-NK-cell signaling, whereas no T-cell-megakaryocyte interaction was detected (**Figure 11C**). Re-clustering of the T-cell compartment identified five subsets: co-stimulatory, naive, cytotoxic, resident, and regulatory T cells (**Figure 12A**).

**Figure 12:**
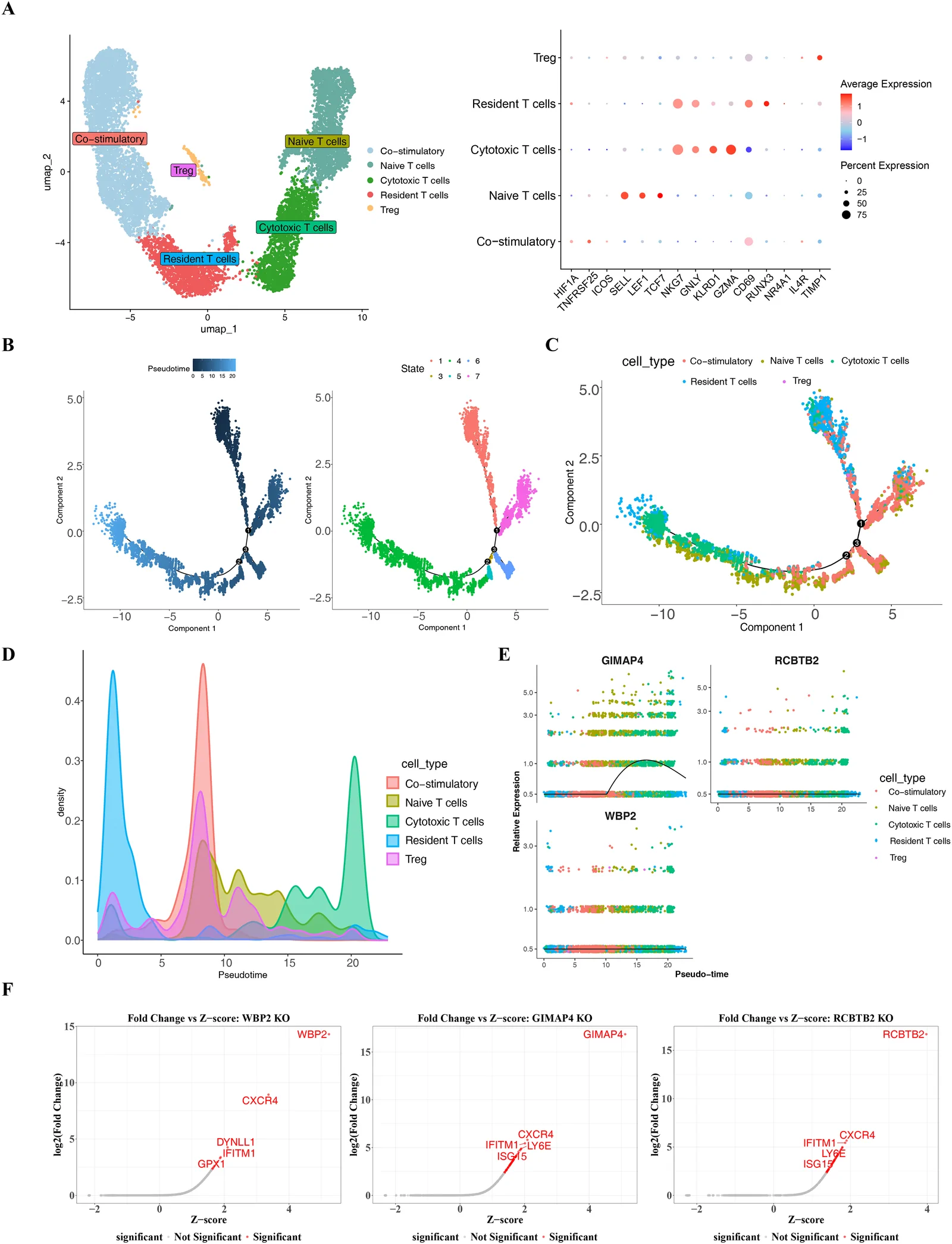
T-cell subtypes, pseudotime trajectories, and virtual gene knockdown. **(A)** UMAP visualization and marker expression of five T-cell subsets. **(B)** Inferred pseudotime and cellular states. **(C)** T-cell subsets distributed along the trajectory. **(D)** Density of each subtype across pseudotime. **(E)** Expression dynamics of *GIMAP4*, *RCBTB2*, and *WBP2*. **(F)** Genes with altered inferred regulation after virtual knockdown of *GIMAP4*, *RCBTB2*, or *WBP2*.

Trajectory analysis assigned the T-cell compartment to six inferred states (**Figure 12B**). State 1 and resident T cells were concentrated near the beginning of the trajectory, whereas state 4 and cytotoxic T cells were more prominent at later positions (**Figures 12C, D**). Among the candidate hub genes and candidate gene requiring validation, only *GIMAP4* changed dynamically during the middle and late portions of pseudotime, increasing initially and then decreasing (**Figure 12E**). Virtual knockdown altered 45 downstream genes for *GIMAP4*, 46 for *RCBTB2*, and 11 for *WBP2*; the five strongest changes for each gene are shown in **Figure 12F**. Overlap of the perturbed sets with SALI DEGs identified *FGL2*, *PLAC8*, *FCGR3A*, *GIMAP4*, *RCBTB2*, *DYNLL1*, *WBP2*, and *GIMAP7* for subsequent investigation (**Figure 12F**).

## 4 Discussion

It should be emphasized that XCD was administered as a whole-formula intervention rather than as a purified cocktail of its individual compounds. The UHPLC-QQQ detection served exclusively for chemical characterization of the decoction, i.e., to identify the major constituents absorbable in rat serum after oral administration. The observed pulmonary protective effects in the animal model are likely attributable to synergistic actions among multiple components of XCD, rather than to any single compound. Although previous studies have reported that rhein, aloe-emodin, rutin, and diosmetin possess anti-inflammatory activities via NLRP3 inflammasome inhibition and NF-κB modulation (50, 51), these findings do not confirm that these specific compounds directly engage the candidate hub genes identified in our multi-omics integration. The multi-component, multi-target nature of XCD warrants further investigation through component knockout/add-back experiments and target engagement assays in future studies. SALI is a critically ill clinical condition with extremely high mortality rates, characterized by uncontrolled inflammatory responses, immune dysfunction, and metabolic imbalance as its core pathological features (4, 52). In recent years, the role of metabolic reprogramming in immune cell function regulation has garnered increasing attention. Peripheral blood serum metabolites can directly and dynamically reflect systemic metabolic alterations during disease states, making them a pivotal research focus for elucidating the pathophysiological mechanisms of SALI and identifying key regulatory molecules. However, studies systematically investigating the associations between metabolites, key genes, and immune cell functions through serum metabolomics-based multi-omics strategies remain relatively limited (53, 54). This study employed integrated metabolomic, transcriptomic and single-cell sequencing analyses to identify oleoylcarnitine as a metabolite signature correlated with SALI, together with GIMAP4, RCBTB2, and WBP2 as candidate hub gene signatures. We further demonstrated stable genetic correlation between these molecular signatures and T-cell populations. Our findings provide multidimensional descriptive insights into SALI molecular profiles, but cannot establish causal pathogenic relationships (55, 56).

This study first screened 34 potential metabolites of XCD intervention on SALI through untargeted metabolomics, and ultimately identified oleoylcarnitine as a candidate serum metabolite supported by GWAS data (57, 58). Our data revealed genetic correlation between oleoylcarnitine-associated polygenic signals and T-cell populations. Together with previous reports that immunometabolic remodeling correlates with immune-cell functional alterations in ALI, our findings suggest oleoylcarnitine may serve as a marker of disturbed fatty-acid oxidation, while we cannot infer a direct causal effect exerted by oleoylcarnitine upon T-cell biology (59, 60). As an important acylcarnitine metabolite, oleoylcarnitine primarily participates in mitochondrial fatty acid oxidation and plays a significant regulatory role in immune cell activation, amplification of inflammatory responses, and tissue damage (61, 62). During lung development, acylcarnitines (including palmitoyl carnitine) exhibit significant dynamic changes and are co-regulated with the expression of carnitine-acylcarnitine translocase (Cact) in lung development (59). Additionally, in patients with septic shock who received L-carnitine supplementation, levels of acylcarnitines (including oleoylcarnitine) increased and remained elevated, with more pronounced elevation observed in non-survivors, which was associated with poor prognosis (63). scPagwas analysis indicated that oleoylcarnitine-related genetic architecture was most strongly associated with T cells, supporting their prioritization for downstream analysis. This finding is also consistent with previous studies indicating that T cell metabolic reprogramming drives sepsis-associated lung injury (64). These findings suggest a potential association between oleoylcarnitine metabolism and T-cell dysfunction in SALI, which requires direct experimental validation. Subsequently, to identify potential key targets at the genetic level, this study employed hdWGCNA to obtain T-cell characteristic genes, combined with differential expression genes and machine learning strategies, ultimately prioritizing GIMAP4 and RCBTB2 as candidate hub genes for SALI, while WBP2 was identified as a candidate gene requiring validation due to inconsistent expression across cohorts. It should be noted that WBP2, despite its contribution to the machine-learning model and functional overlap with GIMAP4 and RCBTB2, did not show significantly altered expression in the validation cohort (GSE32707) or in our independent public validation cohort. This inconsistency may reflect cohort heterogeneity, differences in sample types (whole blood vs. PBMCs), or a smaller effect size that requires larger sample sizes to detect. Therefore, WBP2 should be interpreted with caution and prioritized for future validation studies. Cross-species validation indicated that GIMAP4 is consistently downregulated in human SALI, rat LPS-ALI, and mouse CLP-ALI, providing the strongest cross-species support among the three candidates. RCBTB2 showed human–mouse consistency but not rat consistency. WBP2 did not show stable cross-species changes and is therefore designated as a candidate gene requiring further validation.

Among them, *GIMAP4* is primarily involved in T cell development, homeostasis maintenance, and immune tolerance processes, with its expression closely associated with inflammatory damage and immune dysregulation (65, 66). Previous studies have demonstrated that *GIMAP4* is constitutively expressed in mature T cells and can promote T cell death through endogenous apoptosis pathways, with this effect dependent on its phosphorylation modification (67). Additionally, *GIMAP4* has been identified as a prognostic gene for lung adenocarcinoma (68). Based on these findings, we hypothesize that downregulation of *GIMAP4* expression in SALI may impair T cell apoptosis, leading to abnormal T cell accumulation and hyperactivation, and potentially interact with altered oleoylcarnitine metabolism during SALI progression. *RCBTB2* has been implicated in ubiquitin-mediated protein quality control and immune-related regulation (69). Currently, this gene has been reported in various tumors, such as serving as a methylation-related prognostic gene in lung adenocarcinoma (70) and targeting GPAA1 degradation to inhibit tumor progression in prostate cancer (69). Furthermore, *WBP2*, as a transcription co-activator, can participate in inflammatory signaling pathway regulation (71, 72) and shows an upregulated trend in this study. This gene is a common oncogene that promotes the progression of various tumors, including breast cancer and non-small cell lung cancer (73–75). Neither *RCBTB2* nor *WBP2* has been reported to play a role in SALI.

Based on the SALI background, GSEA analysis was conducted to investigate the functions of key genes, revealing that the three genes were co-enriched in 62 biological processes, notably including innate immune activation and ribonucleoprotein complex subunit organization. Existing studies (60) have demonstrated that during acute lung injury (ALI), the activation of immune cells such as macrophages and lymphocytes is accompanied by significant metabolic reprogramming. This innate immune hyperactivation is intrinsically linked to metabolic reprogramming, as activated macrophages shift toward aerobic glycolysis and fatty acid oxidation to meet the bioenergetic and biosynthetic demands for sustained inflammatory responses-a process that exerts a “double-edged sword” effect by fueling both host defense and collateral tissue damage. Concurrently, the assembly and disassembly of stress granules, which are tightly regulated by ribonucleoprotein complex subunit organization, have emerged as critical checkpoints in innate immune cells during acute inflammatory stress. Stress granules can sequester pro-inflammatory mRNAs and modulate key signaling molecules such as TIA-1 and RIPK1, thereby influencing alveolar epithelial cell and macrophage survival versus apoptosis decisions under septic conditions. Dysregulation of stress granule dynamics has been reported to exacerbate inflammatory cascades in acute lung injury by prolonging the half-life of inflammatory transcripts and promoting necroptotic cell death pathways (76). Together, these two interconnected processes—innate immune activation and ribonucleoprotein complex organization—suggest that the candidate hub genes may participate in SALI pathogenesis through coordinating inflammatory signaling with post-transcriptional regulatory machinery, though this hypothesis requires direct experimental testing.

At the immunological and molecular levels, this study found a significantly elevated proportion of CD56bright natural killer cells in the peripheral blood of SALI patients, while the proportions of eosinophils, macrophages, and myeloid suppressor cells were reduced, indicating a pronounced immune dysregulation state in SALI. Previous studies (77–79) have confirmed that acute septic lung injury is often accompanied by abnormal distribution of peripheral immune cells and coexistence of excessive inflammation and immunosuppression. Abnormal activation of natural killer cells can exacerbate inflammatory cytokine release and lung tissue injury (80), whereas alterations in the proportions of macrophages and eosinophils are closely associated with impaired pulmonary inflammatory clearance capacity (81–84), consistent with the findings of this study. Correlation analysis revealed significant associations between key genes and the aforementioned differential immune cell populations, further supporting their role in immune regulation. Notably, eosinophils, whose infiltration scores positively correlated with RCBTB2 and negatively with WBP2, may link T-cell dysregulation with oleoylcarnitine metabolism: eosinophils exert antigen-presenting and MHC-II-dependent T-cell activating functions in the lung, and their survival and function are highly dependent on fatty acid oxidation (85, 86); thus, oleoylcarnitine-associated metabolic disturbances could impair eosinophil-mediated regulation of T-cell responses via energy deprivation. This interpretation integrates reduced eosinophil abundance into a broader metabolic–immune network involving oleoylcarnitine, T-cell dysfunction, and the candidate genes. Additionally, docking suggested favorable structural compatibility of oleoylcarnitine with GIMAP4 and RCBTB2, but direct binding and functional consequences require biophysical and mutational validation. Subsequently, we have added surface plasmon resonance (SPR) binding assays among recombinant human GIMAP4, RCBTB2 proteins and oleoylcarnitine. The results confirm that oleoylcarnitine binds directly to both human proteins with moderate to high affinity: GIMAP4: KD = 4.16×10^−5^ M; RCBTB2: KD = 1.08×10^−5^ M. These data are consistent with our molecular docking predictions.

Single-cell level analysis revealed significant activation of core metabolic pathways such as the tricarboxylic acid cycle, pyruvate metabolism, and amino acid metabolism in T cells during SALI, indicating substantial metabolic reprogramming of T cells under SALI-induced inflammatory stress. As previously described, T cells in SALI may undergo metabolic reprogramming. Metabolic reprogramming of immune cells, particularly T cells, in sepsis is a critical driver of immune dysregulation, inflammatory amplification, and tissue damage (87–89). Mitochondrial dysfunction and disturbances in glucose and amino acid metabolism further exacerbate pulmonary inflammatory injury (90–92). In both sepsis and SALI groups, T cells functioned as signal-sending populations, with the MIF−(CD74+CXCR4) and MIF−(CD74+CD44) receptor complexes as major inferred interaction axes. Notably, the SALI group specifically exhibited enhanced communication between T cells and NK cells, whereas T-cell–megakaryocyte communication was not detected, reflecting characteristic remodeling of cellular interaction networks during SALI progression. Mechanistically, MIF is a pleiotropic pro-inflammatory cytokine that acts as a key upstream mediator in sepsis and acute lung injury. Upon binding to CD74 receptor complexes (CD74+CXCR4 or CD74+CD44), MIF activates downstream ERK, PI3K/AKT, and NF-κB signaling, promoting macrophage inflammatory polarization and neutrophil recruitment. The enhanced MIF signaling between T cells and NK cells in SALI suggests that these cells may amplify local MIF through paracrine mechanisms, forming a positive feedback inflammatory loop. Notably, MIF inhibitors (e.g., ISO-1, 4-IPP) and anti-MIF antibodies have shown preclinical efficacy in alleviating lung injury, highlighting the MIF-(CD74+CXCR4/CD44) axis as a potential therapeutic target, though strategies specifically targeting T-cell–NK-cell MIF signaling require further validation. Existing studies have confirmed that MIF pathway activation is a central mechanism driving the cascade amplification of sepsis-induced inflammation (93). The significant upregulation of the MIF signaling pathway in the SALI group further suggests more intense and persistently dysregulated pulmonary inflammatory responses. Pseudotime analysis revealed that *GIMAP4* exhibits dynamic expression during the middle and late stages of T cell differentiation. Virtual-knockdown analysis suggested that *GIMAP4*, *RCBTB2*, and *WBP2* can significantly disrupt downstream differential gene expression. In conjunction with the research background on *GIMAP4* family regulation of T cell homeostasis and function, these findings suggest that the aforementioned key genes are indispensable in T cell differentiation and functional maintenance, serving as critical molecules mediating SALI immune dysregulation.

This study used serum metabolites as a starting point and proposed a potential regulatory mechanism of oleoylcarnitine-GIMAP4/RCBTB2/WBP2-T cell dysfunction, providing candidate biomarkers and a mechanistic hypothesis for SALI. However, this study still had several limitations. First, the intratracheal LPS model captures pulmonary inflammation and sepsis-like systemic responses but does not reproduce the polymicrobial sepsis and multi-organ involvement of CLP-induced SALI. Validation of the principal findings in a CLP model was not performed under the present experimental conditions and remains a limitation of this study. Second, oleoylcarnitine was initially derived from serum metabolomics data obtained from the XCD-intervened SALI rat model; all subsequent multi-omics association analyses were entirely based on public human transcriptomic and GWAS datasets (GSE66890, GSE32707, GSE151263). Although we did not directly integrate the rat metabolomic data with the human transcriptomic data, biological heterogeneity still existed between human whole-blood and PBMC transcriptomic profiles due to differences in cellular components. Third, the public datasets used in this study had limited sample sizes, and larger, prospectively collected clinical cohorts were needed. Furthermore, this study relied primarily on bioinformatics analyses and did not conduct gain-of-function or loss-of-function experiments in cellular or animal models, so the inferred regulatory relationships lacked direct experimental evidence. Finally, this study only confirmed at the metabolomic level that XCD reversed oleoylcarnitine-related metabolic alterations in rat serum and did not further verify the specific regulatory effects of XCD on key hub gene expression or T cell function.

## Data Availability

The datasets presented in this study can be found in online repositories. The names of the repository/repositories and accession number(s) can be found below: MTBLS15292 (https://www.ebi.ac.uk/metabolights/MTBLS15292).
